# Targeting the Met-RIPK1 signaling axis to enforce apoptosis and necroptosis in colorectal cancer

**DOI:** 10.1038/s41419-025-08054-5

**Published:** 2025-10-20

**Authors:** Julian Martin Rossmanith, Jennifer Krummeich, Aurel Moser, Kim Lohmann, Michael Linnebacher, Wilfried Roth, Marie Oliver Metzig

**Affiliations:** 1 Institute of Pathology, University Medical Center Mainz, Mainz, Germany; 2 Molecular Oncology and Immunotherapy, Clinic of General Surgery, University Medical Center Rostock, Rostock, Germany

**Keywords:** Cancer therapeutic resistance, Colon cancer

## Abstract

Resistance to cell death remains a critical challenge in the therapy of colorectal cancer (CRC). Smac mimetics (SM) are cytotoxic agents specifically designed to maximize tumor cell killing mediated via endogenous tumor necrosis factor (TNF). In CRC, however, SM lacked clinical activity for reasons that remain incompletely understood. Here, we report that the clinically approved tyrosine kinase inhibitor Cabozantinib potently sensitizes to SM by targeting a Met-RIPK1 signaling axis in CRC. Aberrant Met hampers the activation of RIPK1, which renders CRC cells resistant to TNF/SM-mediated apoptosis and necroptosis. In turn, Cabozantinib potently inhibits Met, thereby stabilizing RIPK1 expression and converting TNF into a robust pro-death signal. SM/Cabozantinib-based regimens demonstrated anti-tumor activity in vivo, and were effective in a heterogeneous panel of patient-derived CRC of diverse molecular subtypes. In addition, we show that it is feasible to modulate between apoptosis and necroptosis to overcome therapy resistance and foster anti-tumor immunity. In summary, this work provides novel biological insight into the mechanisms of SM resistance and warrants the combinatory use of SM and Cabozantinib to enhance apoptotic and necroptotic cell death in CRC.

## Introduction

Colorectal cancer (CRC) is the third most common cancer worldwide [1, 2]. While surgery remains the therapeutic mainstay, adjuvant chemotherapy is offered based on tumor stage and individual risk factors [3]. However, relapse occurs in more than 30% of patients diagnosed in stage II or III, and in 60–70% of patients after metastases resection [3].

Resistance to cell death is a prevalent challenge in CRC and an important reason why chemotherapy fails [4]. Besides targeted strategies to induce apoptosis [5], the discovery of alternative forms of cell death has opened up novel avenues to overcome therapy resistance [6]. Necroptosis is a regulated form of necrosis, which is facilitated when caspase activity - and thus apoptosis - is compromised [6–9]. In addition, necroptosis is an inflammatory form of cell death that may mount an effective anti-tumor immune response [10]. Thus, modulating between apoptosis and necroptosis is a promising strategy to maximize tumor cell killing and improve immune surveillance in cancer [11, 12].

Receptor-interacting protein 1 (RIPK1) is a key regulator of apoptotic and necroptotic cell death [13, 14]. Tumor necrosis factor (TNF) is an inflammatory cytokine commonly present in the microenvironment of tumors [4, 15]. When TNF binds to its receptor (TNFR), RIPK1 is recruited as a scaffold to promote the assembly of a multi-protein complex called complex I [13, 16]. This complex leads to rapid activation of nuclear factor kappa B (NFκB), which induces pro-survival gene expression to protect most types of cancer cells from substantial TNF-mediated cell death [13, 16]. The Inhibitors of apoptosis (IAP) proteins cIAP1 and cIAP2 are pro-survival factors that stabilize RIPK1 in complex I [13, 17]. This constrains RIPK1 kinase activity and hampers the initiation of apoptotic or necroptotic cell death [13, 17]. Smac mimetics (SM) are small-molecule inhibitors designed to target IAP proteins for degradation [18]. By this mechanism, SM are able to unleash RIPK1 kinase activity to foster Caspase-8-mediated apoptosis, or RIPK3-MLKL-mediated necroptosis, respectively [13, 17, 18].

Several SM compounds demonstrated safety and certain anti-cancer potential in clinical trials of treating solid tumors, including CRC [18, 19]. So far, however, SM are not clinically approved, neither as a monotherapy, nor in combination with radiotherapy or other drugs [18]. SM-induced cytotoxicity requires TNF, which may either stem from tumor cells themselves or other cells of the tumor microenvironment (TME) [18, 20, 21]. One of the remaining challenges is that - even in the presence of SM - TNF generates a plethora of pro-survival signals, including NFκB-responsive cIAP2, which is less susceptible to SM-mediated degradation [17, 21]. Thus, in order to harness the potential of SM for clinical use, we need novel strategies to exploit TNF-mediated cytotoxicity, while overcoming concurrent pro-survival signaling feedback [18, 22, 23].

Met is a receptor tyrosine kinase of oncogenic potential, and aberrant activity is frequently reported in CRC [24, 25]. While amplification of the MET gene is a common driver of resistance after anti-EGFR treatment [1, 3, 26], in therapy naïve CRC, Met overexpression and constitutive activation are commonly linked to transcriptional upregulation [27–29]. Cabozantinib is a tyrosine kinase inhibitor with potent activity against Met and clinically approved for the treatment of renal cell carcinoma, hepatocellular carcinoma, and medullary thyroid cancer [30]. While preclinical studies provided evidence that Cabozantinib may also be efficient in CRC, e.g. by suppressing metastasis, angiogenesis and tumor growth [31, 32], it only had limited benefit in clinical trials [26, 33].

Previous reports suggested that MET is a TNF-inducible target gene [34], and that hepatocyte growth factor (HGF), the only known ligand of Met receptor, destabilizes RIPK1 [35]. Thus, we hypothesized that inhibition of Met may facilitate SM/TNF-mediated cell death. Here, we show that indeed Cabozantinib restores SM sensitivity and facilitates apoptotic and necroptotic cell death in CRC.

## Results

### Synergistic effect of Cabozantinib and Smac mimetics (SM) in CRC cells

First, we evaluated the effects of the clinical tyrosine kinase inhibitor Cabozantinib and the SM BV6. We used the human CRC cell line HT29 as a well-established model system to test cytotoxic drug effects in preclinical settings.

**Fig. 1:**
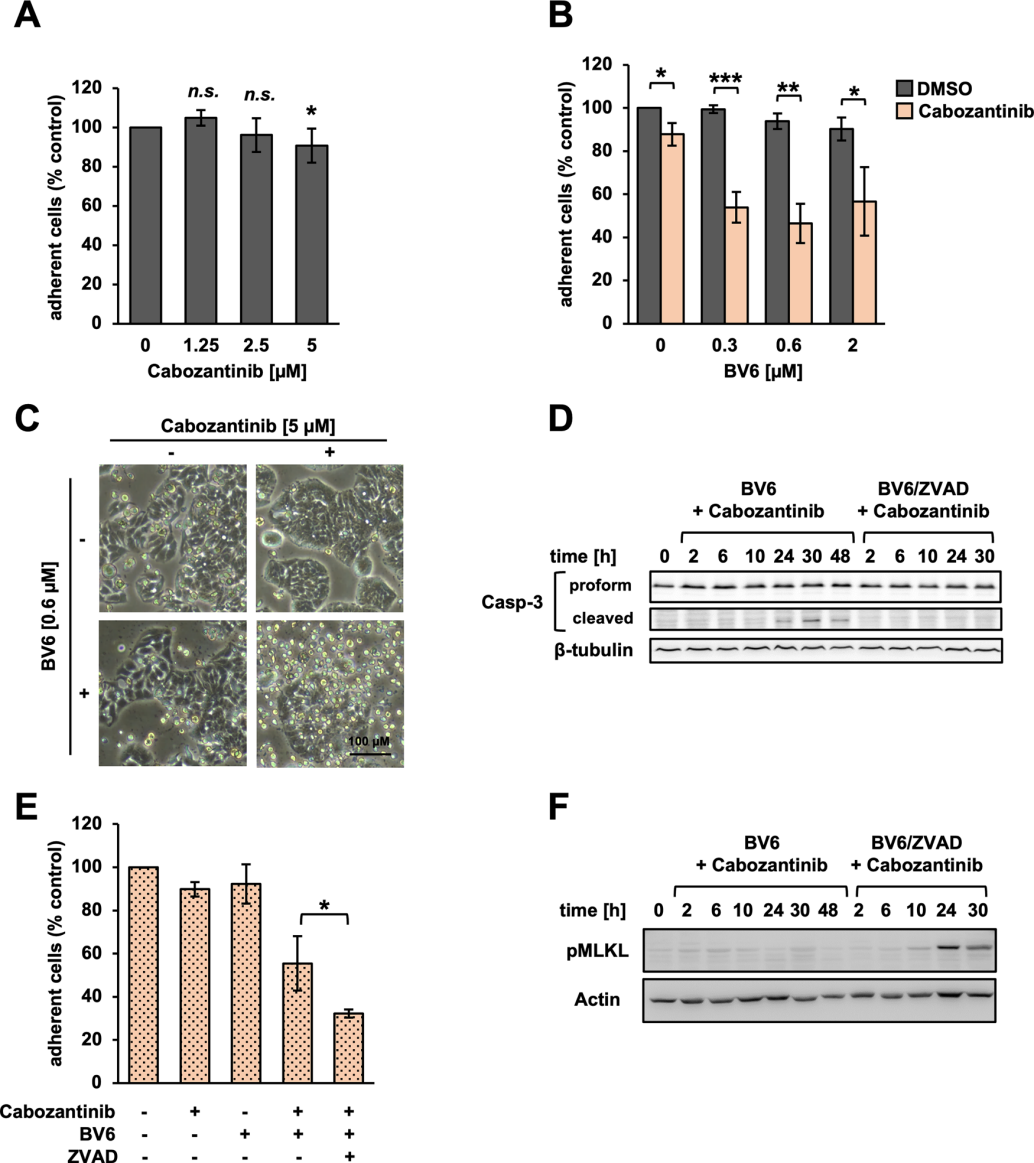
Cabozantinib sensitizes CRC cells for BV6-induced cell death. **A** HT29 cells treated with indicated doses of Cabozantinib for 48 h. Remaining fraction of adherent cells quantified via crystal violet. Mean of three independent experiments ± standard deviation (SD) (*n* = 3). *p* < 0.05 (*), *p* > 0.05 (no significance, n.s.) (Student’s *t* test). **B** HT29 cells pre-treated with Cabozantinib (5 μM for two hours), followed by indicated doses of BV6 for 48 h. Fractional survival quantified via crystal violet. Mean of three independent experiments ± SD (*n* = 3). *p* < 0.05 (*), *p* < 0.01 (**), or *p* < 0.001 (***) (Student’s *t* test). **C** Representative microscopy images illustrating morphological signs of cell death. **D** Western blot for cleaved Caspase-3 in HT29 cells treated with Cabozantinib and BV6 in the presence or absence of pan-caspase inhibitor ZVAD (25 μM, representative data of three independent experiments). **E** Fractional cellular survival after 48 h of treatment with BV6 (0.6 μM), Cabozantinib, or ZVAD. Mean of three independent experiments ± SD (*n* = 3). p < 0.05 (*) (Student’s *t* test). **F** Western blot for pMLKL (representative data of three independent experiments).

Monotherapy with either Cabozantinib or BV6 led to a slight, dose-dependent reduction of the adherent fraction of cells after 48 hours of treatment (Fig. 1A, B). This was in the absence of morphological signs of cell death (Fig. 1C), hinting towards a slight cytostatic effect. Two-hour pre-treatment with Cabozantinib prior to adding BV6, however, led to a marked reduction of adherent cells by up to 54% (Fig. 1B). This was accompanied by rounding up of cells and detachment from the culture plate (Fig. 1C), indicative of cell death. Furthermore, we observed accumulation of cleaved Caspase-3 as a biochemical marker of apoptosis (Fig. 1D), which was abolished by adding the pan-caspase inhibitor ZVAD (Fig. 1D). Interestingly, this led to an even more pronounced reduction of cellular survival (Fig. 1E), along with phosphorylation of MLKL (pMLKL, Fig. 1F), which indicated a switch to necroptotic death.

We concluded that Cabozantinib enables SM-induced apoptosis in CRC cells. Furthermore, it is feasible to controllably push the therapeutic response towards necroptosis by adding pharmacological inhibitors of Caspase activity.

### Cabozantinib-mediated apoptosis and necroptosis depend on RIPK1 and TNF

SM harness the cytotoxic potential of TNF by targeting IAP proteins for degradation [17, 18]. This allows RIPK1 to self-activate and promote the assembly of pro-death complexes that trigger apoptosis or necroptosis, respectively [13].

**Fig. 2:**
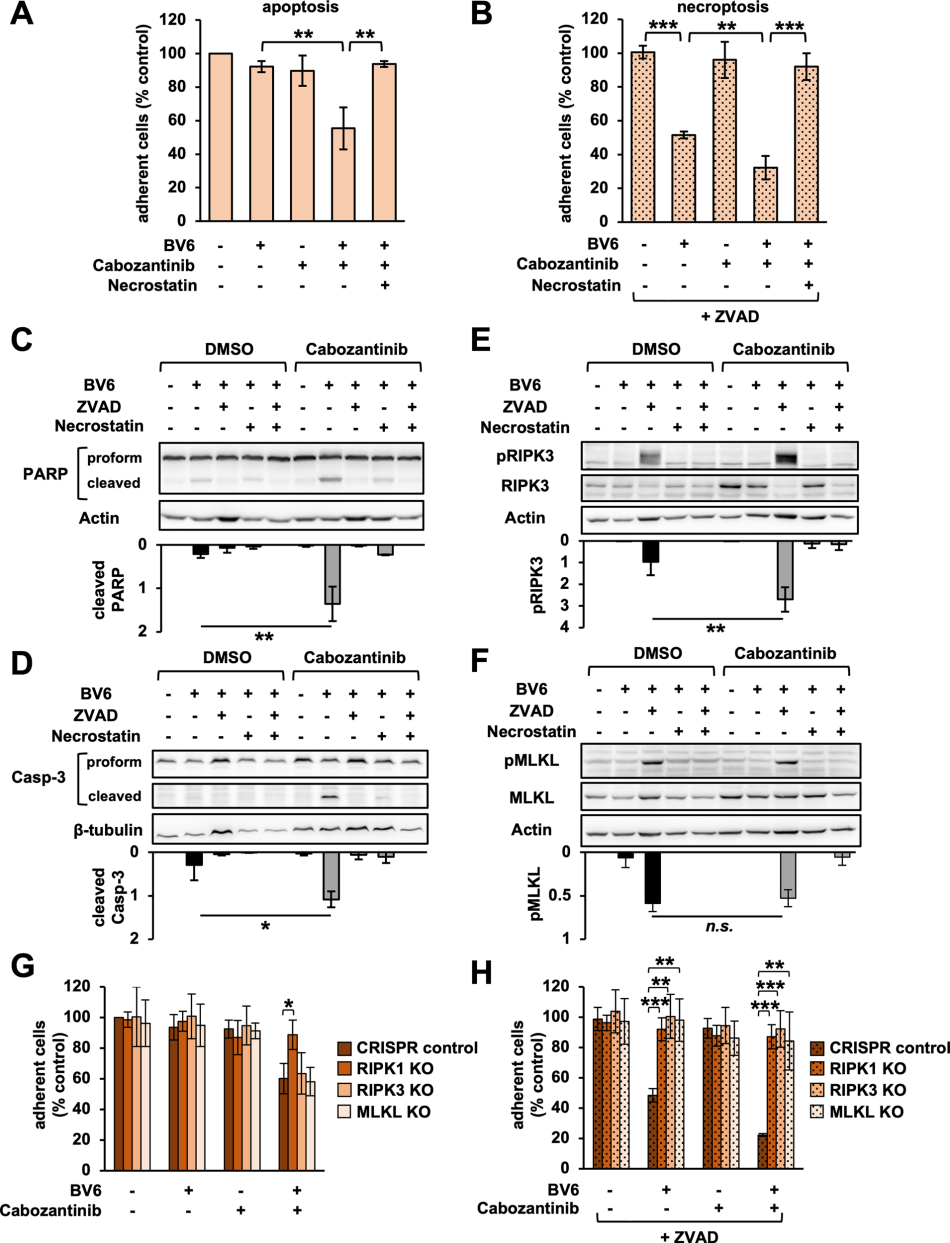
Cabozantinib and BV6 induce apoptotic and necroptotic cell death. **A** HT29 cells treated with Cabozantinib (5 μM) and BV6 (0.6 μM) in the presence or absence of RIPK1 kinase inhibitor Necrostatin-1 (10 μM). Fractional survival after 48 h quantified via crystal violet. Mean of three independent experiments ± standard deviation (SD) (*n* = 3). p < 0.01 (**) (Student’s *t* test). **B** Fractional survival relative to control cells shown in Fig. 2A after 48 h of indicated treatments, here in the presence of ZVAD (25 μM). Mean of three independent experiments ± SD (*n* = 3). *p* < 0.01 (**), *p* < 0.001 (***) (Student’s *t* test).**C**, **D** Western blot for PARP and Caspase-3 in HT29 treated with indicated substances for 30 h. Relative quantification of cleaved protein normalized to proform. Mean of three independent experiments ± SD (*n* = 3). *p* < 0.05 (*), *p* < 0.01 (**), (Student’s *t* test). **E**, **F** Western blot for pRIPK3 and pMLKL in HT29. Relative quantification of phosphorylated normalized to total protein. Mean of three independent experiments ± SD (*n* = 3). *p* > 0.05 (no significance, n.s.), *p* < 0.01 (**), (Student’s *t* test). **G**, **H** Fractional survival in HT29 wild-type (CRISPR control) and CRISPR/Cas9-knockout (KO) cells after 48 h of indicated treatments. Mean of three independent experiments ± SD (*n* = 3). *p* < 0.05 (*), *p* < 0.01 (**), *p* < 0.001 (***) (Student’s *t* test).

Thus, we asked whether the cytotoxic effect of BV6 and Cabozantinib also depended on RIPK1. Indeed, we found that the RIPK1-specific kinase inhibitor Necrostatin-1 restored fractional survival (Fig. 2A, B), and abolished biochemical markers of apoptotic (Fig. 2C, D) and necroptotic cell death in HT29 (Fig. 2E, F). Performing CRISPR-Cas9-mediated knockout confirmed that RIPK1 was required for Cabozantinib/BV6-induced apoptosis and necroptosis (Fig. 2G, H, Supplementary Fig. 1A), while Cabozantinib/BV6/ZVAD-induced necroptosis also depended on RIPK3 and MLKL (Fig. 2H, Supplementary Fig. 1B, C).

**Fig. 3:**
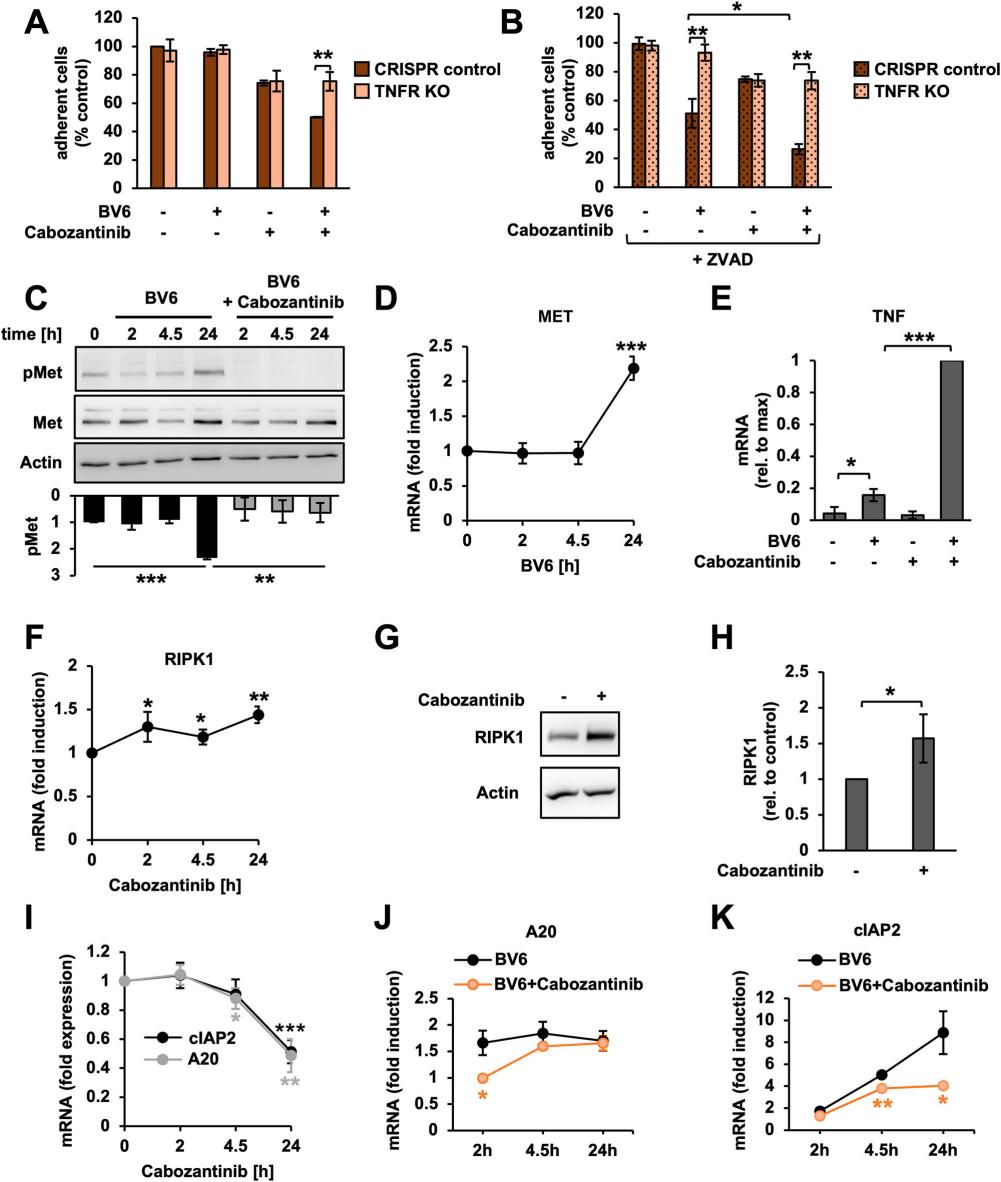
Cabozantinib alleviates Met-mediated block on TNF/RIPK1-dependent cell death. **A**, **B** Fractional survival in HT29 wild-type (CRISPR control) and CRISPR/Cas9-knockout (TNFR KO) cells after 48 h of indicated treatments (BV6: 0.6 μM, Cabozantinib: 5 μM, ZVAD: 25 μM) quantified via crystal violet. Mean of three independent experiments ± standard deviation (SD) (*n* = 3). *p* < 0.05 (*), *p* < 0.01 (**) (Student’s *t* test). **C** Western blot for pMet and Met in HT29 after indicated treatments. Mean of three independent experiments ± SD (*n* = 3). *p* < 0.01 (**), *p* < 0.001 (***) (Student’s *t* test). **D–F** Relative mRNA expression in HT29 after indicated treatments measured via qRT-PCR. Mean of three independent experiments ± SD (*n* = 3). *p* < 0.05 (*), *p* < 0.01 (**), *p* < 0.001 (***) (Student’s *t* test). **G** Western blot for RIPK1 in HT29 treated with Cabozantinib for 24 h (representative data of three independent experiments). **H** Relative quantification of RIPK1 protein in Cabozantinib-treated cells in Fig. 3G. Mean of three independent experiments ± SD (*n* = 3). *p* < 0.05 (*) (Student’s *t* test). **I–K**: Relative mRNA expression measured via qRT-PCR. Mean of three independent experiments ± SD (*n* = 3). *p* < 0.05 (*), *p* < 0.01 (**), *p* < 0.001 (***) (Student’s *t* test).

Previous work established that the cytotoxic effects of SM in CRC require auto- or paracrine TNF [18, 20]. In fact, TNFR-knockout significantly protected HT29 cells from Cabozantinib/BV6-induced apoptosis, and Cabozantinib/BV6/ZVAD-induced necroptosis (Fig. 3A, B, Supplementary Fig. 1D), while adding recombinant TNF accelerated cell death (Supplementary Fig. 1E).

We concluded that Cabozantinib/SM-based apoptosis and necroptosis require RIPK1 and TNF in CRC.

### Met signaling antagonizes the cytotoxic potential of SM

SM and TNF activate concurrent pro-survival pathways that limit RIPK1-mediated cell death [18, 21]. Cabozantinib is a tyrosine kinase inhibitor with potent activity against Met [30]. Given the synergistic effect between BV6 and Cabozantinib, we asked whether Met activation may contribute to SM resistance in CRC.

While moderate levels of phosphorylated Met (pMet, Fig. 3C) in untreated HT29 cells indicated a certain level of basal activity, BV6 led to a significant increase after 24 h of treatment (Fig. 3C). Interestingly, this was in the absence of HGF expression (Supplementary Fig. 2A), the only known ligand of the Met receptor [36]. In fact, MET overexpression, e.g. due to gene amplification or increased protein expression, are known as potential ligand-independent mechanisms of MET activation [28, 29, 36]. Testing this hypothesis we found that BV6 induced the transcription of MET (Fig. 3D), while MET gene amplification is absent in HT29 (Supplementary Fig. 2B–D). Since BV6-induced MET transcription and activation coincided with increased production of TNF mRNA in HT29 (Fig. 3E), we hypothesized that autocrine TNF may be involved. In fact, recombinant TNF led to activation of Met in HT29 cells (Supplementary Fig. 2E), while BV6 failed to induce pMet in TNFR-knockout cells (Supplementary Fig. 2E, F). We concluded that SM leads to MET transcription and activation by mechanisms that involve autocrine TNF.

As expected, co-treatment with Cabozantinib potently blocked basal phosphorylation, and abolished BV6-induced activation of Met (Fig. 3C). Moreover, Cabozantinib significantly enhanced BV6-induced production of autocrine TNF after 24 h of treatment (Fig. 3E, Supplementary Fig. 3A, B), which may facilitate subsequent cell death in HT29. However, since exogenously added TNF alone was insufficient to sensitize cells to BV6 (Supplementary Fig. 3C), we assumed that Cabozantinib enhanced cell death via additional mechanisms.

Previous work associated higher levels of HGF with reduced protein levels of RIPK1 in CRC [35]. In fact, stimulating HT29 with recombinant HGF led to increased activation of Met and reduced RIPK1 protein levels after 24 hours of treatment (Supplementary Fig. 3D, E). In addition, we found that HGF significantly suppressed RIPK1 transcription (Supplementary Fig. 3F), while increasing the expression of cIAP2 and A20 (Supplementary Fig. 3G, H), which are both known to inhibit pro-death functions of RIPK1 [16]. In turn, Cabozantinib significantly enhanced RIPK1 mRNA and protein expression (Fig. 3F–H), and decreased the basal (Fig. 3I) and BV6-inducible expression of A20 and cIAP2 (Fig. 3J, K), while other regulators remained unaffected (Supplementary Fig. 3I, J).

We concluded that while Met activation contributes to SM resistance in CRC, Cabozantinib primes CRC cells for TNF-mediated cell death by stabilizing RIPK1 and dampening the expression of cIAP2 and A20 pro-survival genes.

### Combining Cabozantinib with the clinical substances Birinapant and Emricasan is therapeutically effective

In a next step, we combined Cabozantinib with the clinically relevant substances Birinapant and Emricasan. Birinapant is an SM, which demonstrated safety and certain anti-tumor potential in Phase-2 trials [18]. Emricasan is a pan-caspase inhibitor, which was previously evaluated in Phase-2 trials for hepatitis [37]. Furthermore, Emricasan demonstrated anti-tumor potential in combination with Birinapant [38], or 5-fluorouracil (5-FU) [39] by inducing necroptosis.

Similarly to BV6, HT29 cells were relatively resistant to Birinapant monotherapy (Supplementary Fig. 4A). As expected, Cabozantinib markedly sensitized HT29 cells to Birinapant-induced apoptosis (Supplementary Fig. 4A), which was rescued by Necrostatin-1 (Supplementary Fig. 4B). In line with our previous findings, adding Emricasan switched the response to RIPK1-dependent necroptosis (Supplementary Fig. 4C).

**Fig. 4:**
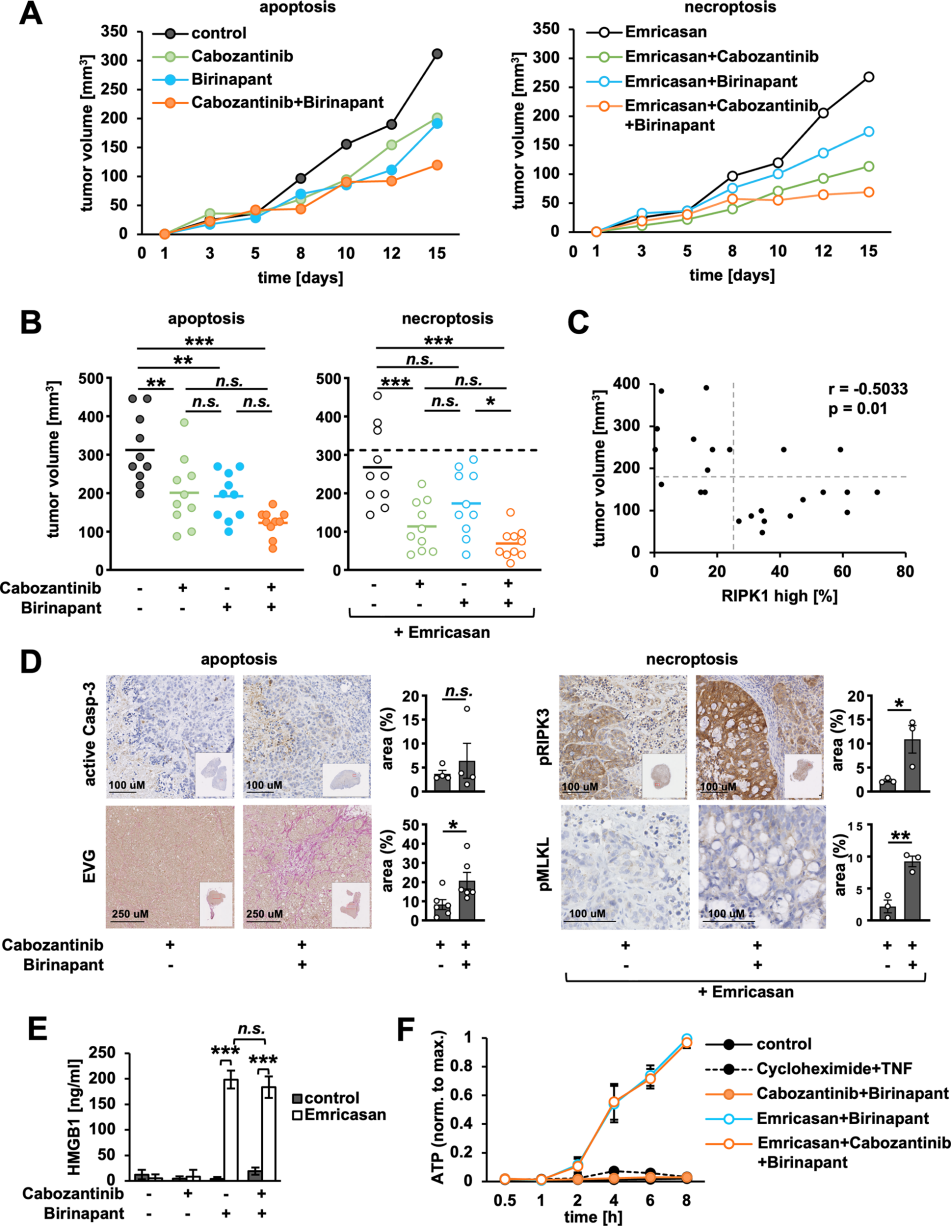
Cabozantinib and the clinical SM Birinapant demonstrate in vivo therapeutic efficacy. **A** HT29 cells were subcutaneously injected into left and right flank of CD1 nude mice. When tumors were palpable (day 0), mice were divided into eight groups with five animals per group to apply apoptotic (left) or necroptotic treatments (right). Every 3 or 4 days, mice received drugs and tumor volumes were measured. Data represent average tumor sizes per group (*n* = 10). **B** Tumor volumes on day 15 for apoptotic (left) and necroptotic treatment groups (right). Each circle represents one individual tumor. Bars are group average, dashed line is average of control group (*n* = 10). p < 0.05 (*), *p* < 0.01 (**), *p* < 0.001 (***) (one-way ANOVA with Bonferroni’s correction). **C** Scatter plot to illustrate inverse correlation between tumor sizes and the percentage of tumor area with high RIPK1 expression (see Supplementary Fig. 4E, and Methods section). Each circle represents one individual tumor (total of *n* = 24, including *n* = 3 per treatment group) (r, Spearman correlation analysis). **D** Immunohistochemical stainings of cleaved Caspase-3, pRIPK3 and pMLKL, or Van Gieson’s Elastica (EVG) staining to quantify fibrotic tumor area in apoptotic (left, *n* = 4) or necroptotic treatment groups (right, *n* = 3). Quantification depicts percentage of positively stained tumor area; each circle represents one individual tumor, while bars represent mean ± standard error of the mean (SEM). *p* > 0.05 (no statistical significance, n.s.), *p* < 0.05 (*), *p* < 0.01 (**) (Student’s *t* test). **E** HMGB1 released by necroptotic HT29 cells after 24 h of treatment. Mean of three independent experiments ± standard deviation (SD) (*n* = 3). p < 0.001 (***) (Student’s *t* test). **F** Time course of ATP release by necroptotic HT29 cells. Cycloheximide/TNF was included as an inducer of non-inflammatory cell death. Mean of three independent experiments ± SD (*n* = 3).

We proceeded by testing Cabozantinib/Birinapant-based apoptotic and necroptotic treatment regimens in an in vivo model of HT29 tumor xenografts. To this end, we injected nude mice subcutaneously with HT29 cells, followed by treatments with Cabozantinib/Birinapant or Emricasan/Cabozantinib/Birinapant, respectively (Fig. 4A). Both treatment regimens resulted in significantly reduced tumor volumes when compared to control (Fig. 4B), while regular health and body weight monitoring (Supplementary Fig. 4D) in the absence of adverse events indicated that drug combinations were generally well tolerated. In line with our mechanistic findings, RIPK1 expression was inversely correlated with tumor sizes (Fig. 4C), and was particularly increased in Cabozantinib-treated tumors (Supplementary Fig. 4E). Importantly, Cabozantinib/Birinapant-treated tumors stained positive for the apoptosis marker active Caspase-3 and showed increased fibrosis as a sign of tumor regression (Fig. 4D, left). In contrast, Emricasan/Cabozantinib/Birinapant-treated tumors stained positive for pRIPK3 and pMLKL (Fig. 4D, right), indicative of necroptosis.

Previous work indicated that necroptotic cells generate inflammatory signals that may enhance anti-tumor immunity [10, 40–44]. Indeed, we found that Cabozantinib/Birinapant-induced necroptotic, but not apoptotic cell death resulted in the release of damage-associated patterns (DAMPs) including HMGB1 and ATP (Fig. 4E, F, Supplementary Fig. 4F). Interestingly, Cabozantinib also significantly decreased PD-L1 expression induced by necroptotic therapy (Supplementary Fig. 4G).

Together, these experiments demonstrate the in vivo safety and anti-tumor efficacy of Cabozantinib/Birinapant-based treatment regimens in CRC. Furthermore, inducing necroptosis by Cabozantinib, Birinapant and Emricasan may raise inflammation and optimize anti-tumor immunity.

### SM sensitivity in patient-derived CRC cell lines

Next, we aimed to test the translational significance of our findings in the context of clinical and molecular heterogeneity of CRC. To this end, we used a cohort of six patient-derived CRC cell lines (HROC, see Table 1 and [45, 46], which were relatively resistant to the standard chemotherapeutic 5-FU (Supplementary Fig. 5A, 5B). Four of the six included HROC cell lines harbored mutations of KRAS or BRAF (see Table 1), which constitute mechanisms of primary resistance to anti-EGFR therapies [1, 47].

**Table 1:** Overview of clinical and molecular features of the HROC cell line cohort.

HROC ID	Age / gender	Colonic region	Staging (UICC)	Grading	Molecular subtype	Mutational status			
						P53	KRAS	BRAF	MET
24	98 y / male	C. ascendens	I	G2	spMSI	wt	wt	mt	wt
39	69 y / male	C. ascendens	IIb	G3	spSTD	wt	wt	wt	wt
40	69 y / male	C. descendens	IIIa	G3	CIMP	mt	mt	wt	wt
46	66 y / male	C. dscendens	IV	G3	spSTD	wt	mt	wt	wt
69	62 y / male	C. ascendens	IIIa	G3	spSTD	mt	wt	wt	wt
87	76 y / female	C. ascendens	IIa	G3	spMSI	mt	wt	mt	wt

Abbreviations: *y* years*; UICC* Union for International Cancer Control. Molecular types of colon carcinoma: *spMSI*, sporadic high-degree microsatellite instable; *spSTD*, sporadic standard type; *CIMP* CpG island methylator phenotype. Mutational status: *wt* wildtype, *mt* mutant.

**Fig. 5:**
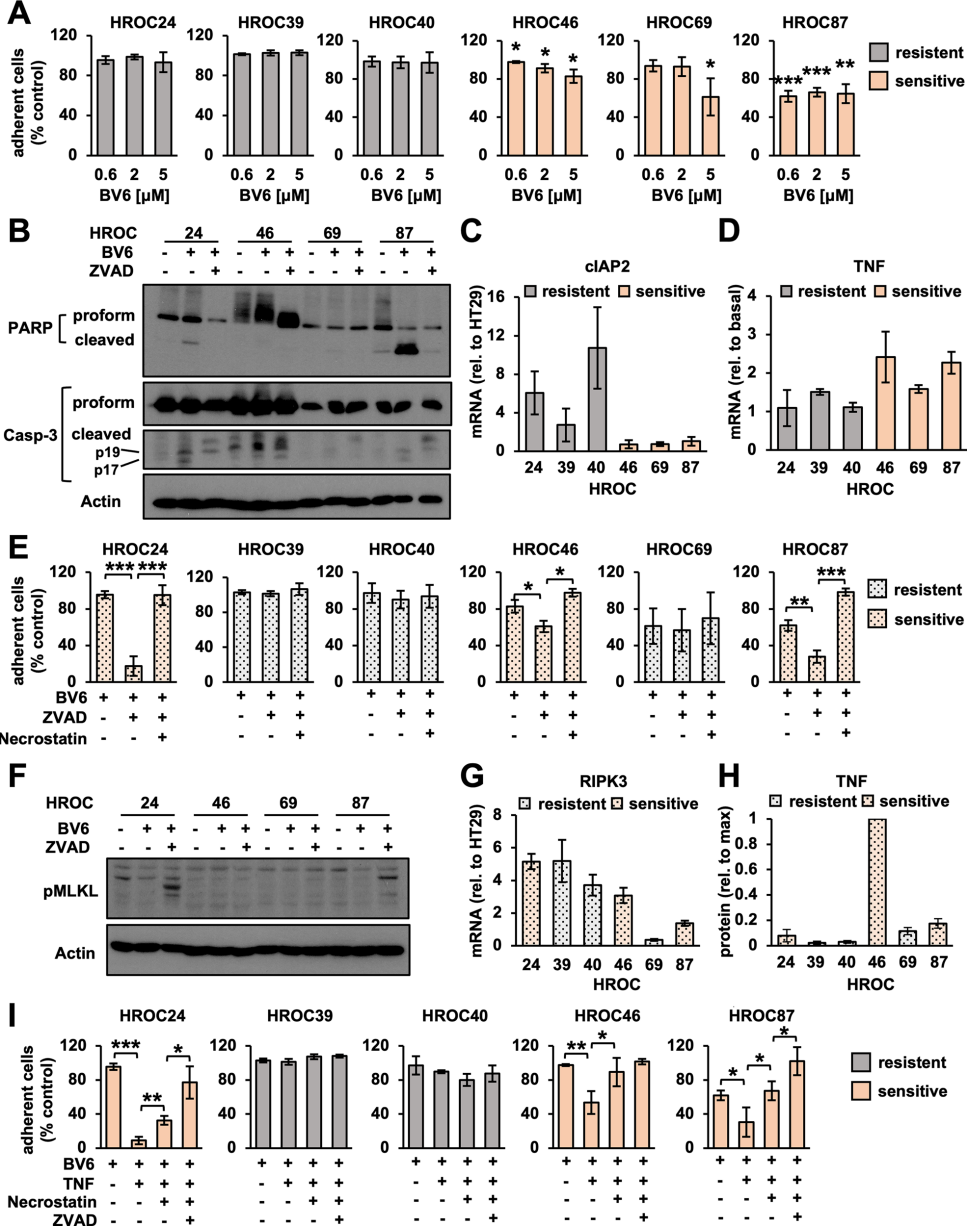
Cytotoxic potential of SM in CRC of heterogeneous clinical and molecular background. **A** Patient-derived HROC cell lines treated with indicated doses of BV6 for 48 h. Fractional survival quantified via crystal violet assay. Mean of three independent experiments ± standard deviation (*n* = 3) (SD). *p* < 0.05 (*), *p* < 0.01 (**), or *p* < 0.001 (***) (Student’s *t* test). **B** Western blot for PARP and Caspase-3 in HROC cells treated with BV6 (HROC24, 87: 0.6 μM; HROC46, 69: 5 μM) and ZVAD (25 μM) for 30 h. **C** Relative mRNA expression measured via qRT-PCR normalized to HT29 as a control. Mean of three independent experiments ± SD (*n* = 3). **D** Fold induction of TNF mRNA expression by BV6 as measured via qRT-PCR. Mean of three independent experiments ± SD (*n* = 3). **E** HROC cell lines treated with BV6 (HROC24, 87: 0.6 μM; HROC39, 40, 46, 69: 5 μM), ZVAD (25 μM) and Necrostatin-1 (10 μM) for 48 h. Fractional survival quantified via crystal violet assay. Mean of three independent experiments ± SD (*n* = 3). *p* < 0.05 (*), *p* < 0.001 (**) or *p* < 0.001 (***). (Student’s *t* test). **F** Western blot for pMLKL in HROC cells treated as indicated in Fig. 5B. **G** Relative expression of RIPK3 mRNA measured via qRT-PCR and normalized to HT29. Mean of three independent experiments ± SD (*n* = 3). **H** Cells treated with BV6 and ZVAD as specified in Fig. 5E. TNF secretion quantified in cell culture supernatants via ELISA, and normalized to HROC46. Means of three independent experiments ± SD (*n* = 3). **I** Cells treated as specified in Fig. 5E, in the presence or absence of TNF (25 ng/ml). Fractional survival quantified via crystal violet assay. Mean of three independent experiments ± SD (*n* = 3). *p* < 0.05 (*), *p* < 0.01 (**) or *p* < 0.001 (***) (Student’s *t* test).

While three cell lines (HROC24, 39 and 40) were resistant to increasing doses of BV6 (Fig. 5A), the three residual cell lines (HROC46, 69 and 87) responded with a mild to moderate reduction of adherent cell numbers (Fig. 5A). Particularly in HROC87 cells, this effect was accompanied by cleavage of PARP and Caspase-3, indicative of apoptotic cell death (Fig. 5B). Resistant cell lines showed relatively high expression levels of cIAP2 (Fig. 5C), but not cIAP1 (Supplementary Fig. 5C), and lacked production of autocrine TNF in response to BV6 (Fig. 5D), which is in line with previous literature [21].

Adding ZVAD, however, sensitized HROC24 cells to BV6, and also facilitated necroptotic cell death in HROC46 and HROC87 cells (Fig. 5E, F), while HROC69 did not respond due to lack of RIPK3 expression (Fig. 5G) [20]. In contrast, the RIPK3-positive cell lines HROC39 and HROC40 remained resistant (Fig. 5E), despite substantial expression of RIPK1 and TNFR (Supplementary Fig. 5D), or substitution of recombinant TNF (Fig. 5H, I).

In summary, the cytotoxic potential of SM varied amongst chemotherapy-resistant CRC of diverse molecular subtypes. Interestingly, RIPK3 expression was widely preserved, but insufficient to ensure successful execution of necroptosis. As previously suggested, therapeutic efficacy partially depended on the expression of cIAP2, the availability of TNF, and likely additional factors that rendered certain CRC cell lines resistant toward SM.

### Cabozantinib overcomes therapy resistance in patient-derived CRC

Based on our mechanistic findings in the HT29 model, we hypothesized that SM resistance was linked to aberrant Met activation in the HROC cohort.

**Fig. 6:**
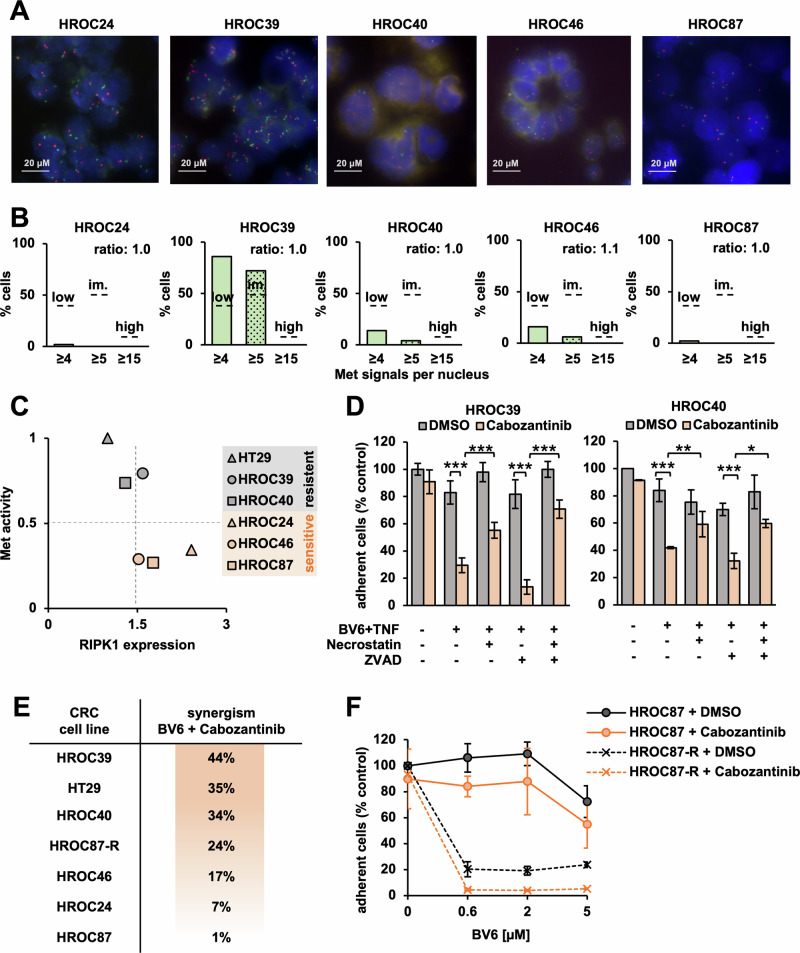
Cabozantinib overcomes therapy resistance in CRC of diverse molecular backgrounds. **A** FISH hybridization of probes against the MET gene (green) and centromere 7 (orange) to HROC nuclei (blue). **B** Status of MET gene amplification in HROC cells. Count of MET signals across 50 nuclei revealed intermediate-level (im) amplification in HROC39 cells. MET/centromere ratio was < 2 in all tested cell lines (cutoffs indicated by dashed lines and according to diagnostic guidelines,(58) see details in Methods section). **C** Scatter plot to illustrate inverse correlation between basal levels of pMet (see Supplementary Fig. 6C, D) and RIPK1 expression (Supplementary Fig. 6E) in indicated cell lines. **D** HROC39 (left) or HROC40 (right) treated with BV6/TNF, Cabozantinib (5 μM), ZVAD (25 μM) and Necrostatin-1 (10 μM) for 48 h. Fractional survival quantified via crystal violet assay. Mean of three independent experiments ± standard deviation (SD) (*n* = 3). *p* < 0.05 (*), *p* < 0.01 (**), *p* < 0.001 (***) (Student’s *t* test). **E** Loss of fractional survival in CRC cell lines due to synergism between BV6 and Cabozantinib in the presence of TNF (based on Fig. 6D, Supplementary Fig. 6F–I). Synergism is implied if combination produces a greater reduction of fractional survival than additive effects of single drugs. **F** Fractional survival of parental cell line HROC87 and derivative cell line HROC87-R, which is resistant to Encorafenib and Cetuximab.(48) Mean of three independent experiments ± SD (*n* = 3).

As expected, most HROC cell lines did not harbor MET gene amplification (Fig. 6A, B) and MET mutations were absent (see Table 1). HROC39, however, which was one of the most resistant cell lines that neither responded to 5-FU, nor to combinations of SM, TNF, or ZVAD (Fig. 5, Supplementary Fig. 5A, B), showed intermediate-level amplification of the MET gene (Fig. 6A, B) in the absence of HGF expression (Supplementary Fig. 6A). Furthermore, HROC39 cells in particular showed elevated levels of basal and BV6-induced pMet similar to HT29 cells (Supplementary Fig. 6B–6D).

Since we previously associated active Met signaling with decreased expression of RIPK1, we compared basal Met activity (Supplementary Fig. 6C, D) and RIPK1 mRNA levels (Supplementary Fig. 6E) across all tested CRC cell lines. Interestingly, this analysis implied an inverse correlation between Met activity and RIPK1 expression (Fig. 6C). In particular, the BV6/TNF-resistant cell lines HROC39 and HROC40 harbored relatively high levels of pMet and low expression levels of RIPK1 (Fig. 6C).

Importantly, adding Cabozantinib sensitized both HROC39 and HROC40 cells for BV6/TNF-induced apoptotic, or BV6/TNF/ZVAD-induced necroptotic cell death, respectively (Fig. 6D). Other tested HROC cell lines also responded to Cabozantinib/BV6 at varying degrees (Supplementary Fig. 6F–6I) with synergistic effects ranging between 44% and 1% (Fig. 6E). Strikingly, Cabozantinib/BV6-based therapy was also effective in a derivative of the BRAF-mutant cell line HROC87 with acquired resistance to BRAF- and EGFR-targeting agents (HROC87-R [48], Fig. 6F).

Together, our data support that targeting the Met/RIPK1 circuit via Cabozantinib enhances the sensitivity towards SM-induced apoptosis and necroptosis. Our data further imply that this could demonstrate a vital option for patients with CRC of diverse clinical and molecular backgrounds, in which standard therapies, including 5-FU or anti-EGFR, fail. Thus, our study warrants the further clinical evaluation of SM in combination with Cabozantinib to overcome therapy resistance in CRC.

## Discussion

Despite improvements in the adjuvant treatment of patients with advanced CRC, therapy resistance, relapse and poor survival remain common challenges [49]. Novel personalized strategies are urgently needed to overcome apoptosis resistance and trigger an effective anti-tumor immune response.

TNF is a key inflammatory cytokine, which was originally identified for its potential to induce tumor necrosis [15]. However, systemic application as an anti-cancer agent is impossible, due to the risk of cytokine storm and subsequent shock [50]. SM are anti-cancer agents specifically designed to harness the killing potential of endogenous TNF, which is produced by tumor cells itself and other cells of the TME [18]. Despite high safety and tolerability, however, SM have not lived up to expectations [18]. In clinical trials for treating solid tumors, including CRC, limited cytotoxic activity was partially attributed to the activation of concurrent pro-survival signals [18, 21]. Drug-induced NFκB, for instance, mediates the activation of gene expression programs that hamper the initiation and execution of cell death, and foster therapy resistance [4, 18, 21, 51].

**Fig. 7:**
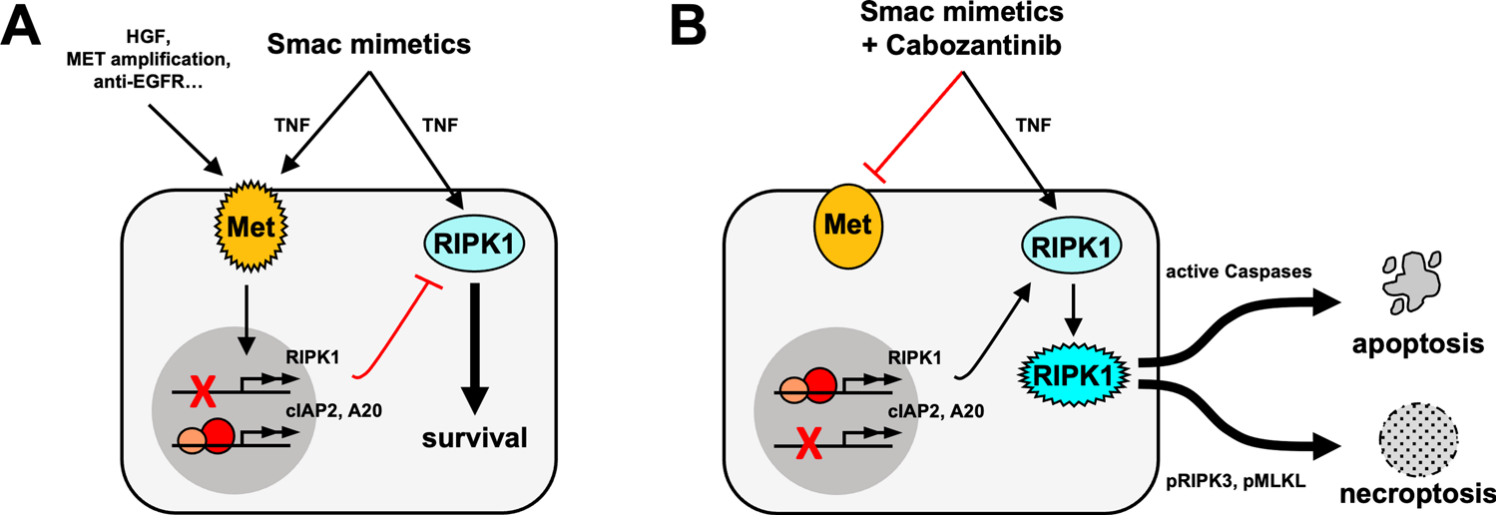
Schematics of Cabozantinib targeting the Met-RIPK1 axis to overcome SM resistance in CRC. **A** Genetic or epigenetic misregulation lead to aberrant Met activity. Met suppresses RIPK1 expression and increases TNF-responsive pro-survival genes, e.g., cIAP2 and A20, to inhibit SM-induced cell death. **B** Cabozantinib blocks Met activity, which stabilizes RIPK1 expression and dampens TNF-induced pro-survival gene expression programs to unleash RIPK1 kinase and trigger SM-induced cell death. Sufficient activation of caspases will license apoptosis, while necroptosis is favored in RIPK3-positive tumors with compromised Caspase activity.

Here, we report that resistance to SM in CRC is linked to an additional mechanism encoded by the Met-RIPK1 signaling axis. We show that aberrant Met due to prior drug therapy, genetic or epigenetic aberrance decreases RIPK1 expression and renders CRC cells resistant to TNF-mediated cell death (Fig. 7A). In turn, the clinical tyrosine kinase inhibitor Cabozantinib blocks Met activation, stabilizes RIPK1 expression, and dampens the expression of SM resistance genes cIAP2 and A20 to convert TNF into a robust pro-death signal (Fig. 7B).

Since Cabozantinib is a multi-target tyrosine kinase inhibitor [30], we cannot exclude that other mechanisms besides Met inhibition play a role. In particular, previous studies highlighted blockage of VEGFR, which leads to disrupted vasculature in solid tumors [30, 32, 52]. In fact, besides the induction of apoptotic and necroptotic cell death this mechanism may have likely contributed to the in vivo effects we observed in Cabozantinib-treated tumors (Fig. 4).

Cabozantinib is approved for the therapy of several tumors, but so far has not been effective in CRC [26, 30, 33]. Although MET gene amplification is a key driver of anti-EGFR resistance in CRC, Cabozantinib monotherapy or in combination with Panitumumab had only limited anti-tumor activity [26, 30, 33, 53]. Strikingly, our data imply that—when combined with SM—Cabozantinib is effective in CRC of diverse subtypes, including scenarios of primary (e.g., KRAS- or BRAF-mutations), and acquired resistance (e.g., aberrant MET). Thus, SM/Cabozantinib-based regimens may be an urgently needed alternative, in particular for patients in which conventional chemotherapy with 5-FU, or anti-EGFR therapies currently fail.

Furthermore, our study demonstrates that it is feasible to switch between death modalities and induce necroptosis in CRC, e.g., by adding the clinical pan-caspase inhibitor Emricasan [37, 39, 54]. This strategy was effective under standardized in vivo conditions (Fig. 4), and in the context of diverse CRC subtypes (Fig. 5). Interestingly, five out of the six tested patient-derived CRC cell lines expressed substantial levels of RIPK3, which is a crucial pre-requisite for necroptotic cell death [20]. This is in contrast to widely used immortalized cell lines, in which silenced expression of RIPK3 is a common finding [20, 55]. Although notoriously challenging to work with, this underlines the importance of translational studies that include patient-isolated cell lines, which adhere more closely to the clinical and molecular features of CRC [45, 46].

What might be the clinical benefit of modulating between apoptotic and necroptotic cell death? On the one hand, necroptosis particularly occurs under Caspase-deficient conditions, and thus is an important backup strategy in apoptosis-resistant CRC [39]. On the other hand, previous work indicated that necroptosis is more inflammatory than apoptosis, possibly due to concordant activation of NFκB [40–44]. This is in line with our finding that Cabozantinib/SM-based necroptosis, but not apoptosis, leads to the release of DAMPs. In fact, by this mechanism, necroptosis may enhance antigen-presentation and mount a pre-existing tumor-specific T-cell response [11, 12], a pre-requisite for immunotherapy [56]. Today, immune checkpoint inhibitors are ineffective in the vast majority of CRC patients [49, 57]. In this context, the future clinical value of combining SM with Cabozantinib may extend beyond simply maximizing cell death towards optimizing anti-tumor immunity, and priming for successful immunotherapy.

## Materials and methods

Key reagents and resources as listed in Table 2.

**Table 2:** Key reagents and resources.

REAGENT or RESOURCE	SOURCE	IDENTIFIER
*Antibodies*		
Caspase-3	Novus Biologicals	NB100-56708
beta-Tubulin	Cell Signaling	2128
phospho-MLKL	Abcam	ab187091
Actin	Merck	MAB1501
PARP	BD Biosciences	556362
Vinculin	Cell Signaling	4650
phospho-RIPK3	Abcam	ab209384
RIPK1	Cell Signaling	3493
RIPK3	Novus Biologicals	NBP2-24588
MLKL	Cell Signaling	14993
Met	Cell Signaling	3127
phospho-Met	Cell Signaling	3077
cleaved Caspase-3	Cell Signaling	9579S
anti-Rabbit HRP conjugate	BioRad	170-6515
anti-Mouse HRP conjugate	BioRad	170-6516
Biotinylated anti-rabbit IgG (H + L)	Vector Laboratories	BA-1000
*Chemicals, peptides and recombinant proteins*		
Cabozantinib	Absource Diagnostics	S1119
BV6	Genentech, Inc.	
DMSO	Sigma-Aldrich	D2650
Z-VAD(OMe)-FMK	Enzo Life Sciences	BML-P416
Necrostatin-1	Enzo Life Sciences	BML-AP309
Human recombinant TNF	R&D	210-TA
HGF	Thermo Fisher Scientific	100-39H
Birinapant	Absource Diagnostics	S7015
Emricasan	Absource Diagnostics	S7775
Captisol	CYDEX Pharmaceuticals	RC-0C7-020
5-FU	Sigma-Aldrich	F6627
Super Signal West Pico Plus	Thermo Fisher Scientific	34580
WesternBright Sirius	Biozym	541021
Cell Lysis Buffer (10X)	Cell Signaling	9803
Halt Protease and Phosphatase Inhibitor Cocktail	Thermo Fisher Scientific	78440
DUB inhibitor PR-619	Sigma-Aldrich	662141
OPTI-MEM medium	Gibco	31985-062
UltraCULTURE medium	Lonza	BP12-725F
Lipofectamine 2000	Thermo Fisher Scientific	11668-019
Crystal violet	Neofroxx	LC 7034.2
RNeasy Plus Mini Kit	Qiagen	74134
SYBR Green PCR MasterMix	Thermo Fisher Scientific	4364346
High Capacity cDNA Reverse Transcription Kit	Applied Biosystems	4368813
ZytoLight FISH-Tissue Implementation Kit	ZytoVison	Z-2028-20
ZytoLight SPEC MET/CEN 7 Dual Color Probe	ZytoVison	Z-2087-200
QIAamp DNA Mini Kit	Qiagen	51306
Lumit® HMGB1 Human/Mouse Immunoassay	Promega	W6110
RealTime-Glo™ Extracellular ATP Assay	Promega	GA5010
Human TNF ELISA Kit Quantikine	R&D	DTA00D
Dako EnVision FLEX HRP/Dab	Agilent	K8010
Dako EnVision Flex Target Retrieval Solution	Agilent	DM829
*Plasmids*		
lentiCRISPRv2	Addgene	52961
pMDLg/pRPE	Addgene	12251
pRSV-Rev	Addgene	12253
pMD2.G	Addgene	12259
Cell lines		
HT29	ATCC	
HROC 24	Michael Linnebacher	
HROC 39	Michael Linnebacher	
HROC 40	Michael Linnebacher/Cytion	#30082
HROC 46	Michael Linnebacher	
HROC 69	Michael Linnebacher	
HROC 87	Michael Linnebacher	
HROC 87-R	Federica Di Nicolantonio	
Organisms/strains		
CD1 nude mice (Crl:CD1-Foxn1^nu^)	Charles River	
One Shot Stbl3 chemically competent E. coli	Thermo Fisher Scientific	C737303
Software and algorithms		
Microsoft Excel 2016	Microsoft Office	
Graphpad prism 9	Graphpad Software, Inc.	
Fusion Solo 6 and Evolution Capt software 17.04a	Vilber Lourmat	
QuPath 5.0	Bankhead et al. [61]	
QuantStudio Design & Analysis Software v1.5.1	Applied Biosystems	
NIS-Elements v5.20.00	Nikon	
SparkControl v3.1	Tecan Group	
Other		
gRNA sequences, see Table S1		
Primer sequences, see Table S2		

### Experimental model and subject details

#### Cell culture

The human CRC cell line HT29 (ATCC) was maintained in RPMI-1640 medium (Gibco, 21875-034) containing 10% FBS (Sigma-Aldrich, F7524) and 1% penicillin/streptomycin (Sigma-Aldrich, P0781) at 5% CO_2_ and 37 °C. Patient-derived low-passage HROC cell lines (provided by PD Dr. Michael Linnebacher and Cytion) [45, 46] were kept in DMEM medium (Gibco, 41966-029), supplemented with 20% FBS, 1% Glutamax (Gibco, 35050-038) and 1% penicillin/streptomycin at 5% CO_2_ and 37 °C. The HROC87 derivative cell line with resistance to Encorafenib and Cetuximab (HROC87-R, kindly provided by Federica Di Nicolantonio) was generated as previously described [48]. All cell lines were regularly confirmed to be free of mycoplasma contamination using MycoAlert Mycoplasma Detection Kit (Lonza, LT07-318) and MycoAlert Assay Control Set (Lonza, LT07-518).

#### Mouse experiments

All animal work in accordance with the NIH guidelines Guide for the Care and Use of Laboratory Animals. Animal protocol was approved by the German animal welfare authorities (LUA RLP, permit number: G22-1-100). 4 to 5 weeks old female CD1 nude mice (Crl:CD1-Foxn1^nu^) were obtained from Charles River, and housed under specific-pathogen free conditions with regular health monitoring.

### Method details

#### Cell viability assay

HT29 or HROC cells were seeded in 96-well plates (20,000 or 30,000 cells per well, respectively) and grown for 24 h. Cells were pre-treated with Cabozantinib (5 μM), ZVAD (25 μM), Emricasan (25 μM) and/or Necrostatin-1 (10 μM) for 2 h, followed by adding SM (BV6 or Birinapant) and/or TNF (25 ng/ml). After 24 or 48 h, remaining adherent cells were stained with crystal violet (0.5% in 20% methanol for 10 min), washed with water and sodium citrate solution (0.1 M in 50% ethanol) was added prior to measuring absorbance using Tecan Spark microplate reader (Tecan Group). Data were normalized to mock-treated controls. Data analysis was performed using Tecan Spark Control Software (Tecan Group) and Microsoft Excel 2016. Microscopic pictures were taken with Nikon Eclipse Ts2 using Series Color Microscope Camera (The Imaging Source, DFK 33UX264). Surrogate markers of inflammatory cell death were quantified in cell culture supernatants via Lumit® HMGB1 Human/Mouse Immunoassay (Promega), and RealTime-Glo™ Extracellular ATP Assay (Promega), respectively [10]. Luminescent detection and data analysis was performed using Tecan Spark microplate reader. Data analysis was done using Tecan Spark Control Software and Microsoft Excel 2016.

#### Western blotting

Cells were grown in 6 cm dishes for 24 h and treated as indicated. Dead cells were removed by washing with ice-cold PBS. Remaining adherent cellular fraction was lysed in lysis buffer (Cell Signaling, 9803) supplemented with protease and phosphatase inhibitor and de-ubiquitinase inhibitor PR-619 (Sigma-Aldrich). Samples were normalized for total protein using a Bradford assay (Bio-Rad), boiled for 2 min in 1x SDS sample buffer, and subjected to gel electrophoresis and immunoblotting. Following blocking and antibody incubation, a signal was developed using Super Signal West Pico Plus (Thermo Fisher Scientific) or WesternBright Sirius (Biozym), detected using Fusion Solo S imaging system (Vilber Lourmat) and relatively quantified with Evolution Capt software (Vilber Lourmat). Full and uncropped Western blot images are provided in Supplemental Material. Data analysis was performed in Microsoft Excel 2016.

#### CRISPR/Cas9-gene editing

Guide RNAs (gRNAs, Table S1) were cloned into lentiCRISPR v2 (Addgene) [58], and transfected into HEK293T alongside with packaging plasmids to produce lentivirus. Infected HT29 cells were selected with Puromycin (1 µg/ml) until cell death subsided, and used for experiments as indicated. Knockout was confirmed by Western blot or high-resolution melt (HRM) analysis. To this end, genomic DNA was isolated using QIAamp DNA Mini Kit (Qiagen), subjected to PCR amplification (5′ – GCCCTGGCTGTTGTCCCTAG – 3′, and 5′ – TCCTGCCTGTGCACACTCAC – 3′), and subsequent melt curve analysis using SYBR Green (Thermo Fisher Scientific) with temperature increments (0.2°C steps).

#### Quantitative real-time PCR (qRT-PCR)

RNA was purified using RNeasy Plus Mini Kit (Qiagen). cDNA was synthesized with High Capacity cDNA Reverse Transcription Kit (Applied Biosystems). qRT–PCR was performed with SYBR Green PCR Master Mix and QuantStudio 3 Real-Time PCR System (Thermo Fisher) using the D(DCt) method with Actin as normalization control, relative to unstimulated and stimulated signals in HT29 or HROC cells to derive fold change (primer sequences listed in Table S2). Analysis was performed using QuantStudio Design & Analysis Software (Applied Biosystems) and Microsoft Excel 2016.

#### ELISA

Cell culture supernatants were collected from 6-cm dishes, and centrifuged at 1500 rpm for 10 min at 4 °C to remove dead cells. TNF was quantified using Quantikine ELISA kit (R&D Systems) and Tecan Spark microplate reader (Tecan Group). Data analysis was performed using Tecan Spark Control Software (Tecan Group) and Microsoft Excel 2016.

#### Fluorescence in-situ hybridization (FISH)

Cells were harvested from 10 cm dishes, washed in ice cold PBS, centrifuged, and fixed in buffered 4% formalin solution for 15 min at 37 °C. Cells were resuspended in ethanol, immobilized with FBS, and embedded in paraffin. Proteolysis was performed on de-paraffized histological sections (4 μm) using Zyto Light FISH-Tissue Implementation Kit (ZytoVison), and denaturation and hybridization was carried out using ZytoLight SPEC MET/CEN 7 Dual Color Probe (ZytoVison) [59]. To detect MET amplification, cells were imaged with a fluorescence microscope with double-pass filter (Olympus, Japan) using a 60x objective. For analysis, green (MET) and orange (centromere 7, CEN7) signals were counted in 50 nuclei per CRC cell line. MET/CEN7 ratio, average MET copy number per cell, and the percentage of tumor cells with low-level ( ≥4 signals), intermediate-level ( ≥5 signals), or high-level amplification ( ≥15 signals) were calculated to define positivity according to established cutoffs [60]. Data analysis was performed with Microsoft Excel 2016.

#### Animal studies

Six-week-old female athymic CD1 nude mice (Charles River, Germany) were injected subcutaneously with 1 × 10^6^ HT29 cells in 200 μl PBS in the right and left flank using a 25-gauge needle. As soon as tumors were palpable animals were randomly divided into eight groups of treatment (*n* = 10) receiving the following substances: control group (PBS), Birinapant (7.5 mg/kg/week), Cabozantinib (90 mg/kg/week), or Emricasan (3.75 mg/kg/week), or dual and triple combinations as indicated in Fig. 4. Cabozantinib diluted in PBS was given via oral gavage, while Birinapant and Emricasan (formulated in 6% Captisol in PBS) were applied via intraperitoneal injection. Tumor volumes were measured using a micrometer and the ellipsoid formula (length x width x height x $1/2$). At endpoint, tumors were fixed in 4% buffered formalin and embedded in paraffin to obtain histological sections for subsequent analysis. Data analysis to compare tumor volumes was performed using GraphPad Prism 9.

#### Histology, immunohistochemistry and digital pathology

Immunohistochemical stainings of cell death markers in HT29 xenograft tissues was performed using Dako EnVision FLEX Kit (Agilent, K8010), primary antibodies against RIPK1 (1:150), cleaved Caspase-3 (1:250), phospho-RIPK3 (1:500), and phospho-MLKL (1:500), and biotinylated anti-rabbit IgG (Vector Laboratories). Slides were digitalized (40x, Nano Zoomer, Hamamatsu) and analyzed using QuPath software. Percentage of vital tumor area with high RIPK1 expression was quantified using an appropriate threshold (0.25). Percentage of total tumor area staining positive for active Caspase-3, pRIPK3 and pMLKL was quantified using an appropriate (threshold 0.2). Fibrosis as a parameter of tumor regression was visualized by Van Gieson’s Elastica (EVG) staining, and percentage of fibrotic tumor area was quantified by using the QuPath Pixel classifier.

### Quantification and statistical analysis

Details regarding the statistical analyses, including the number of biological replicates, are provided in the figure legends. Routinely, statistical analyses were carried out with unpaired two-tailed Student’s *t* test using Microsoft Excel 2016, or with one-way ANOVA using GraphPad Prism 9 as specified in the figure legends, with *p* < 0.05 (*), *p* < 0.01 (**), and *p* < 0.001 (***) considered significant. Spearman correlation analysis between tumor sizes and RIPK1 expression was carried out using GraphPad Prism 9. Error bars represent standard deviation (SD), or standard error of the mean (SEM) as specified in the figure legends.

## Supplementary information

Supplemental Figure and Supplemental Table Legends

Original data

Supplemental Figure 1

Supplemental Figure 2

Supplemental Figure 3

Supplemental Figure 4

Supplemental Figure 5

Supplemental Figure 6

Supplemental Table S1

Supplemental Table S2

## Data Availability

All data included in the main and supplemental materials. This paper does not report original code. Any additional information required to reanalyze the data reported in this paper is available from the lead contact upon request.

## Materials availability

This study did not generate new unique reagents.

## Code availability

All data are included in the main and supplemental materials. This paper does not report original code. Any additional information required to reanalyze the data reported in this paper is available from the lead contact upon request.
