# Supplementary material for: Targeting the Met-RIPK1 signaling axis to enforce apoptosis and necroptosis in colorectal cancer
Source: Cell Death Dis. 2025 Oct 20;16(1):733. doi: 10.1038/s41419-025-08054-5 (PMC12537949; doi:10.1038/s41419-025-08054-5)
Supplement: Supplementary file 1 — Supplemental Figure and Supplemental Table Legends [file 41419_2025_8054_MOESM1_ESM.docx]

**SUPPLEMENTAL FIGURE LEGENDS**

**Supplemental Figure 1**

**A - C**: Western blot for RIPK1, RIPK3 and MLKL in HT29 wild-type (CRISPR control) and CRISPR/Cas9-knockout (KO) cells.

**D**: CRISPR/Cas9-mediated knockout of TNFR validated via high-resolution melt (HRM) analysis.

**E**: Fractional survival of HT29 wild-type cells treated with BV6 (0.6 μM), Cabozantinib (5 μM) and/or TNF (25 ng/ml) for 24 or 48 hours. Quantification via crystal violet assay. Mean of three independent experiments ± standard deviation (SD) (n = 3). p > 0.05 (no statistical significance, n.s.), p < 0.001 (***) (Student’s *t* test).

**Supplemental Figure 2**

**A**: Relative mRNA expression of HGF measured via qRT-PCR. Human liver samples served as a control. HT29 cells left untreated or were treated with BV6 (0.6 μM) for 24 hours. Mean of three independent experiments ± standard deviation (SD) (n = 3).

**B**: FISH hybridization of probes against the MET gene (green) and centromere 7 (orange) to HT29 nuclei (blue).

**C**: Average count of signals per nucleus across 50 nuclei revealed MET/centromere ratio of 0.9. Mean ± SD (n = 50).

**D**: Status of MET gene amplification in HT29. Counts were below cut-offs for low-, intermediate- or high-level amplification (dashed lines; see Method details section).

**E**: HT29 cells were treated with TNF (25 ng/ml), or with BV6 (0.6 μM) and Cabozantinib (5 μM). Western blot for pMet and Met in HT29 wild-type (CRISPR control) and TNFR-knockout (KO) cells (representative data of three independent experiments).

**F**: Relative quantification of pMet in Supplemental Figure 2E. Mean of three independent experiments ± SD. p < 0.05 (*), p > 0.05 (no statistical significance, n.s.) (Student’s *t* test).

**Supplemental Figure 3**

**A**: HT29 cells treated with ZVAD (25 μM), BV6 (0.6 μM) and/or Cabozantinib (5 μM) for 24 hours. Relative mRNA expression measured via qRT-PCR. Mean of three independent experiments ± standard deviation (SD) (n = 3). p < 0.05 (*), p < 0.001 (***) (Student’s *t* test).

**B**: TNF secretion analyzed in cell culture supernatants from Supplemental Figure 3A via ELISA. Mean of three independent experiments ± SD (n = 3). p < 0.01 (**), p < 0.001 (***) (Student’s *t* test).

**C**: Fractional survival of HT29 cells treated with BV6 and/or TNF (25 ng/ml) for 48 hours as quantified via crystal violet assay. Mean of three independent experiments ± SD (n = 3). p > 0.05 (no statistical significance, n.s.) (Student’s *t* test).

**D**: HT29 cells were treated with HGF (50 ng/ml) for indicated durations. Western blot for pMet and RIPK1.

**E**: Relative quantification of RIPK1 protein after 24 hours of HGF treatment in Supplemental Figure 3D. Mean of three independent experiments ± SD (n = 3). p < 0.05 (*) (Student’s *t* test).

**F**: Relative mRNA expression in response to HGF as measured via qRT-PCR. Mean of three independent experiments ± SD (n = 3). p < 0.05 (*), p < 0.01 (**) (Student’s *t* test).

**G, H**: HT29 cells treated with HGF (50 ng/ml) for indicated durations. Relative mRNA expression measured via qRT-PCR. Mean of three independent experiments ± SD (n = 3). p < 0.01 (**), p < 0.001 (***) (Student’s *t* test).

**I, J**: Relative mRNA expression measured via qRT-PCR. Mean of three independent experiments ± SD (n = 3). p < 0.05 (*), p > 0.05 (no significance, n.s.). (Student’s *t* test).

**Supplemental Figure 4**

**A**: HT29 cells treated with Cabozantinib (5 μM) and/or the SM Birinapant for 48 hours. Fractional survival quantified by crystal violet assay. Mean of three independent experiments ± standard deviation (SD) (n = 3). p < 0.05 (*), p < 0.001 (***) (Student’s *t* test).

**B**: HT29 cells treated with Cabozantinib or Birinapant (0.6 μM) to induce apoptosis in the presence or absence of RIPK1 kinase inhibitor Necrostatin-1 (10 μM). Fractional survival after 48 hours quantified via crystal violet. Mean of three independent experiments ± SD (n = 3). p < 0.001 (***) (Student’s *t* test).

**C**: HT29 cells treated with indicated drugs in the presence of the clinical pan-caspase inhibitor Emricasan (25 μM) to induce necroptosis. Fractional survival after 48 hours relative to control cells shown in Supplemental Figure 4B quantified via crystal violet. Mean of three independent experiments ± SD (n = 3). p < 0.01 (**), p < 0.001 (***) (Student’s *t* test).

**D**: Average body weight of mice harboring HT29 tumor xenografts for the indicated treatment groups. Mean ± SD (n = 5).

**E**: Immunohistochemical stainings of RIPK1 in HT29 xenograft tumors receiving apoptotic (left) or necroptotic treatments (right). Quantification depicts percentage of vital tumor area with RIPK1 staining above the threshold (see Methods section). Each circle represents one individual tumor, while bars represent mean ± standard error of the mean (SEM). Statistical significance was calculated between treatments with or without Cabozantinib (n = 6). p > 0.05 (no statistical significance, n.s.), p < 0.05 (*) (Student’s *t* test).

**F**: Fractional survival of HT29 cells treated with indicated substances for 4 or 8 hours quantified via crystal violet. CHX/TNF was included as a control for non-inflammatory cell death. Mean of three independent experiments ± SD (n = 3). p < 0.05 (*), p < 0.01 (**), p < 0.001 (***) (Student’s *t* test).

**G**: Relative mRNA expression measured via qRT-PCR. Mean of three independent experiments ± SD (n = 3). p < 0.01 (**), p < 0.001 (***) (Student’s *t* test).

**Supplemental Figure 5**

**A**: HT29 and HROC cells treated with indicated doses of the standard chemotherapeutic 5-fluorouracil (5-FU). Fractional survival after 48 hours quantified via crystal violet. Mean of three independent experiments ± standard deviation (SD) (n = 3). p > 0.05 (no statistical significance, n.s.), p < 0.05 (*), p < 0.01 (**), p < 0.001 (***) (Student’s *t* test).

**B**: Summary of 5-FU activity in indicated cell lines. IC50 doses (based on Supplemental Figure 5A) were approximately 2- to 30-fold higher in HROC cell lines compared to HT29 (except for HROC24).

**C, D**: Relative basal expression of mRNA in HROC cell line cohort measured via qRT-PCR and normalized to HT29. Mean of three independent experiments ± SD (n = 3).

**Supplemental Figure 6**

**A**: Relative mRNA expression of HGF measured via qRT-PCR. Human liver samples served as a control. HROC cells were treated with BV6 (0.6 μM) for 24 hours. Mean of three independent experiments ± standard deviation SD (n = 3).

**B**: Western blot for pMet and Met in HT29 and HROC cells treated with BV6 (0.6 μM) and TNF (25 ng/ml).

**C**: Western blot of basal pMet and Met in indicated cell lines.

**D**: Relative quantification of basal pMet in Supplemental Figure 6C (normalized to HT29). Mean of three independent experiments ± SD (n = 3).

**E**: Relative quantification of basal RIPK1 mRNA measured via qRT-PCR in indicated cell lines (normalized to HT29). Mean of three independent experiments ± SD (n = 3).

**F - I**: HROC cell lines treated with Cabozantinib (5 μM), BV6 and/or TNF (25 ng/ml) for 48 hours. Fractional survival compared to untreated quantified by crystal violet assay. Mean of three independent experiments ± SD (n = 3). p < 0.05 (*), p < 0.01 (**), p < 0.001 (***) (Student’s *t* test).

**SUPPLEMENTAL TABLE LEGENDS**

**Table S1: List of used gRNA sequences.**

**Table S2: List of used primer sequences.**
