# Supplementary material for: Targeting the Met-RIPK1 signaling axis to enforce apoptosis and necroptosis in colorectal cancer
Source: Cell Death Dis. 2025 Oct 20;16(1):733. doi: 10.1038/s41419-025-08054-5 (PMC12537949; doi:10.1038/s41419-025-08054-5)
Supplement: Supplementary file 2 — Original data [file 41419_2025_8054_MOESM2_ESM.pdf]

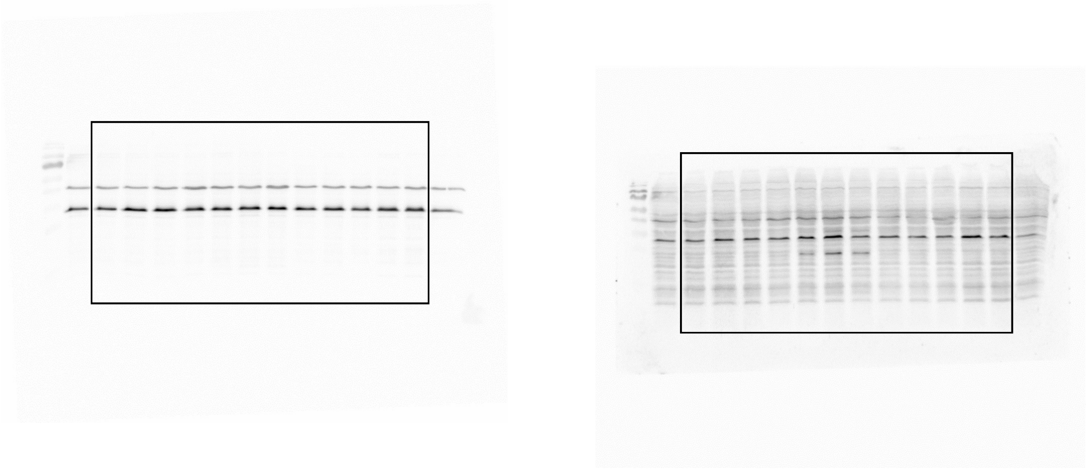

Figure 1D

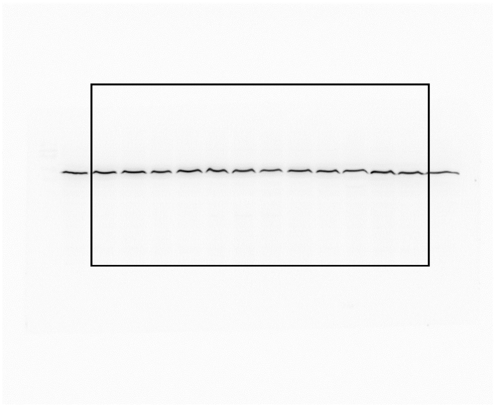

Figure 1F

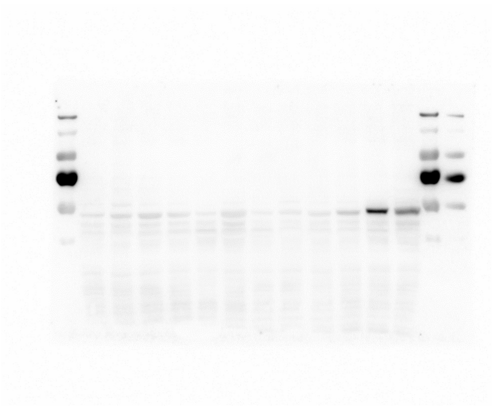

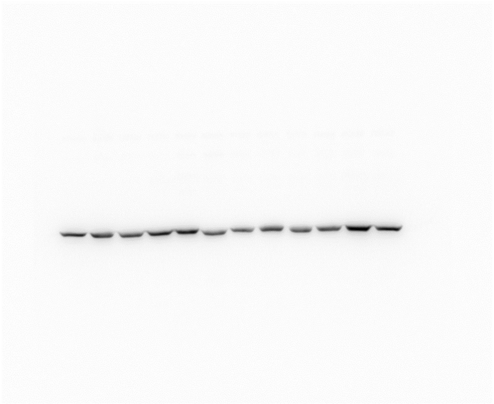

Figure 1F

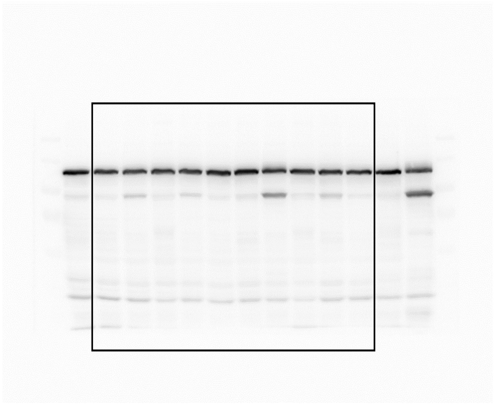
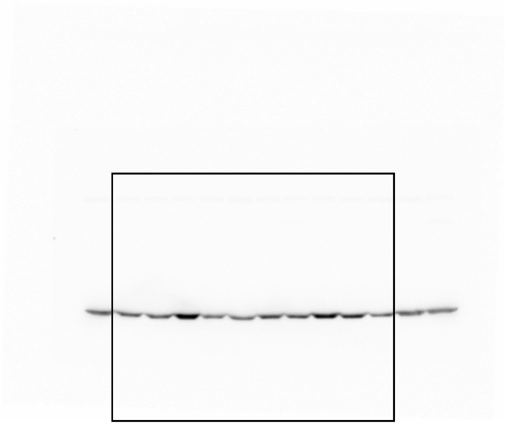

Figure 2C

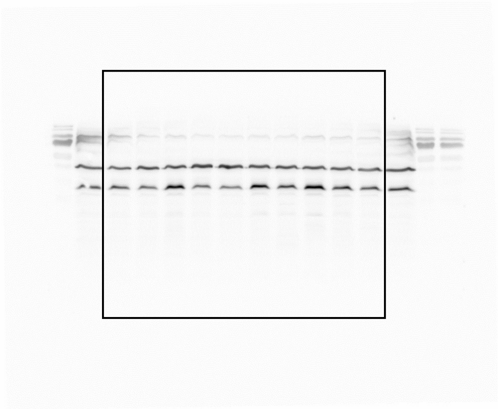

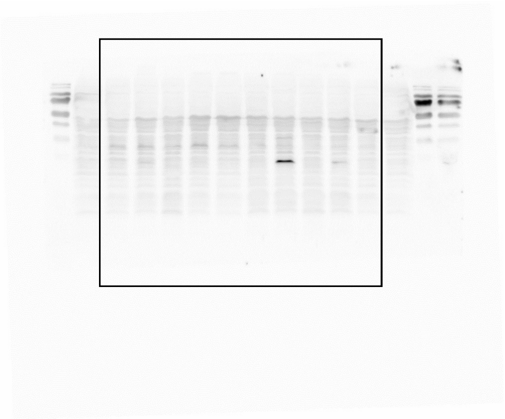

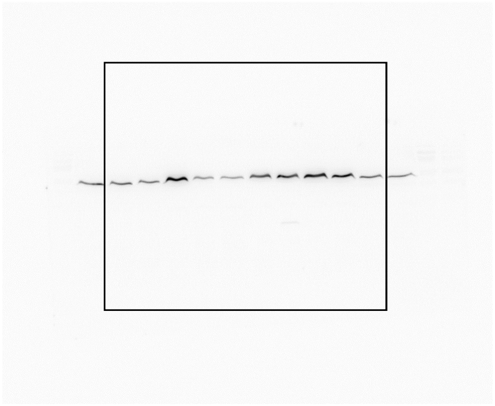

Figure 2D

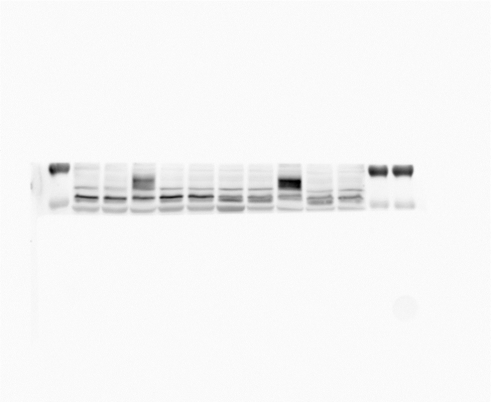

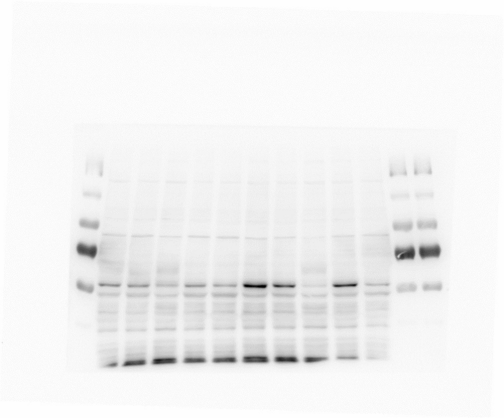

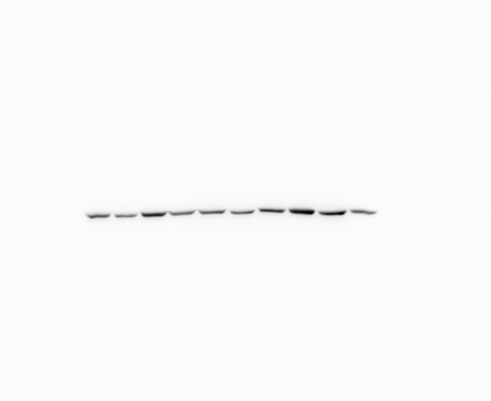

Figure 2E

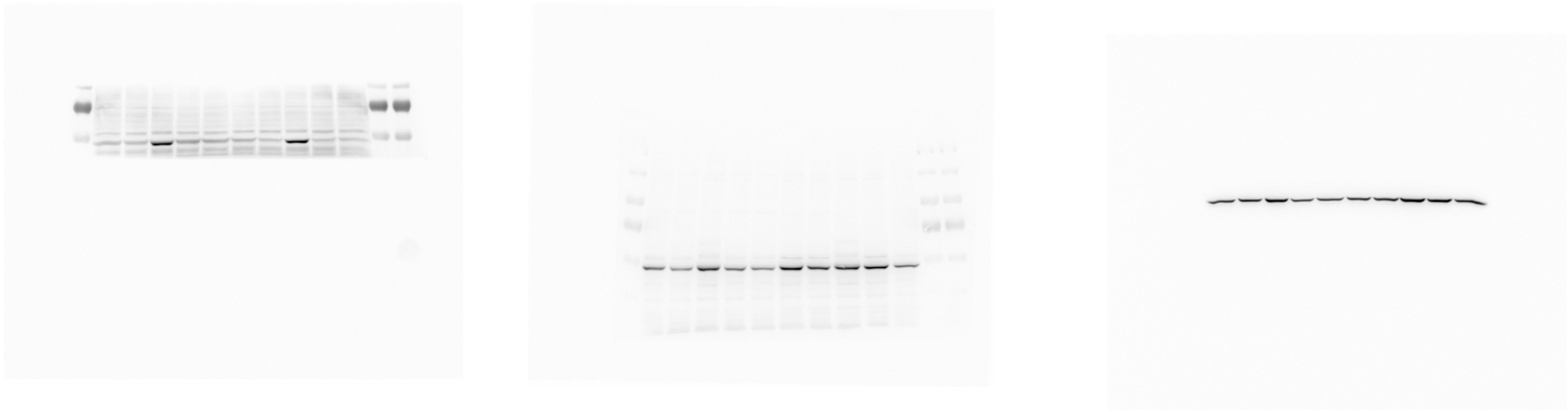

Figure 2F

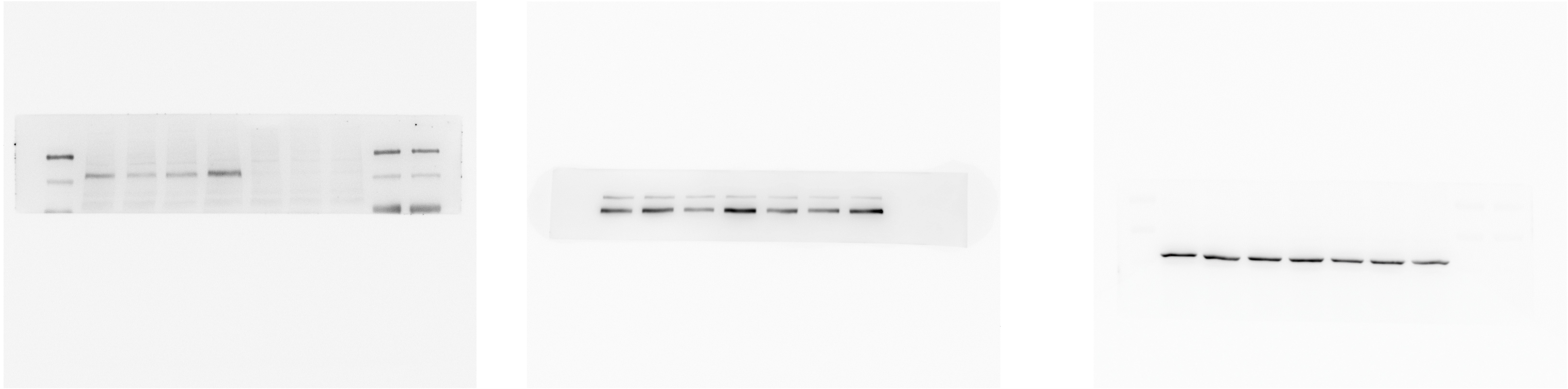

Figure 3C

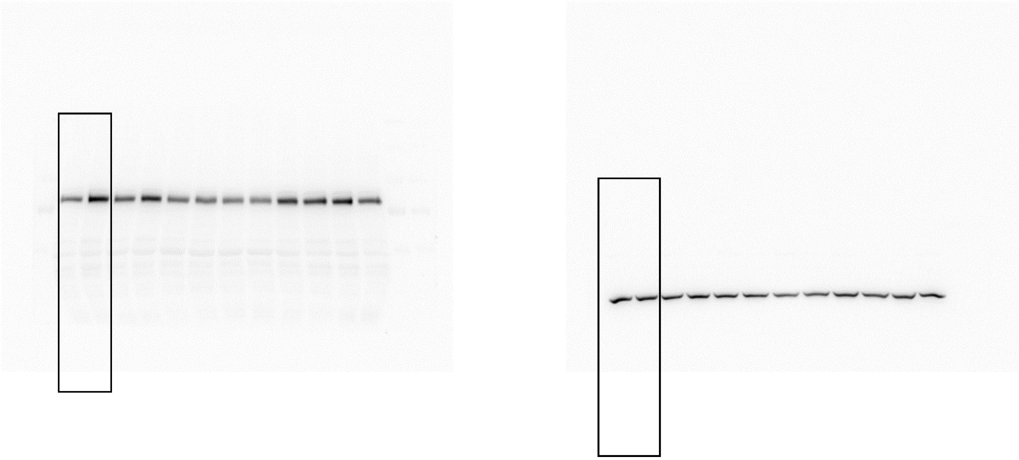

Figure 3G

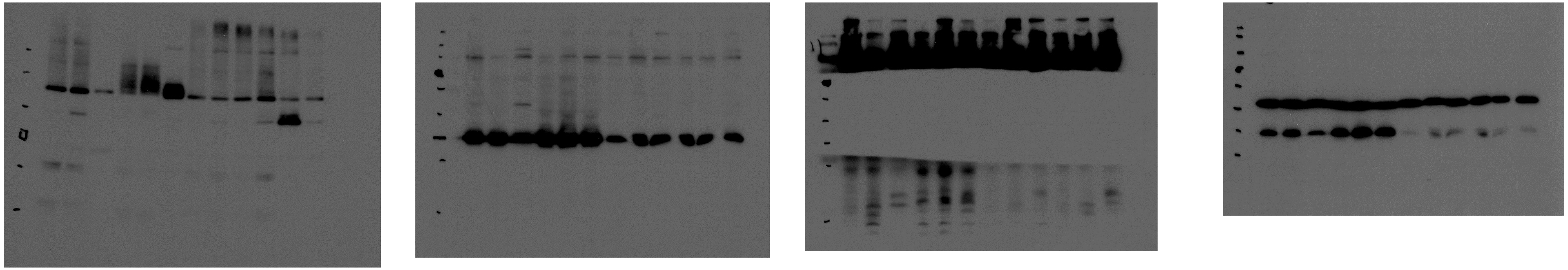

Figure 5B

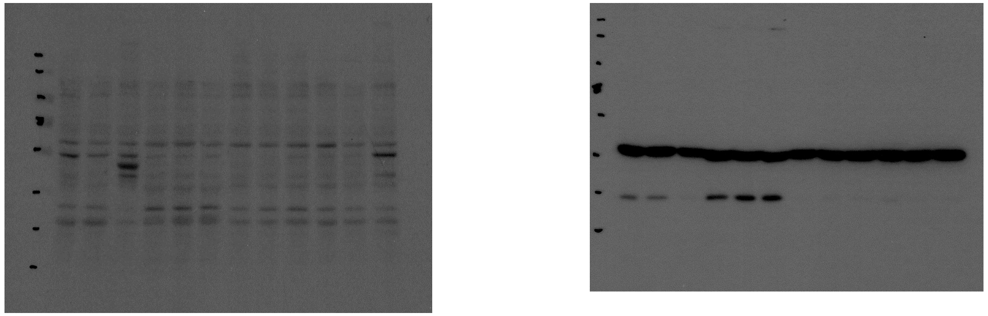

Figure 5F

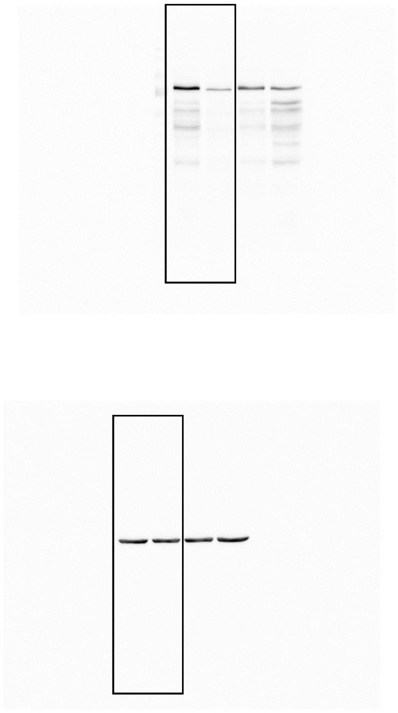

Supplemental Figure 1A

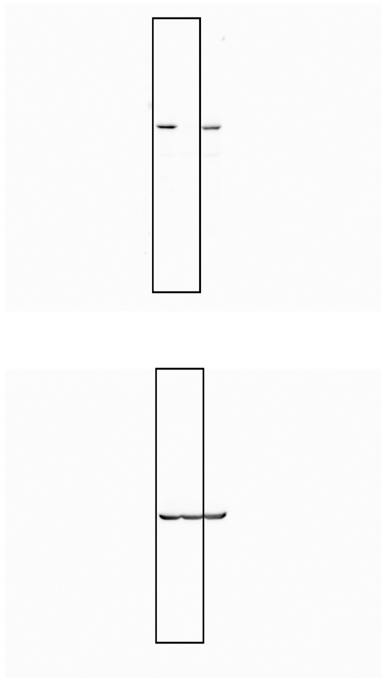

Supplemental Figure 1B

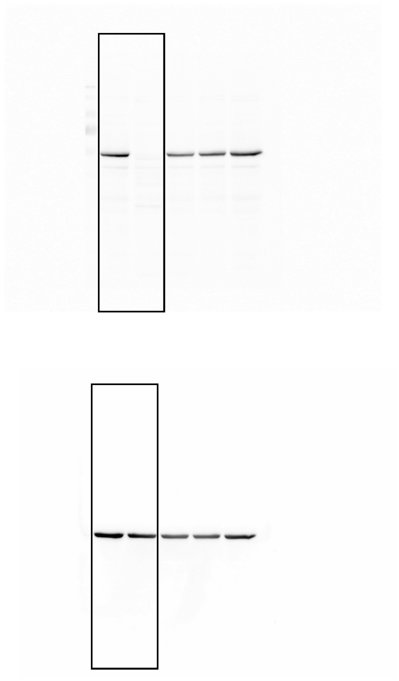

Supplemental Figure 1C

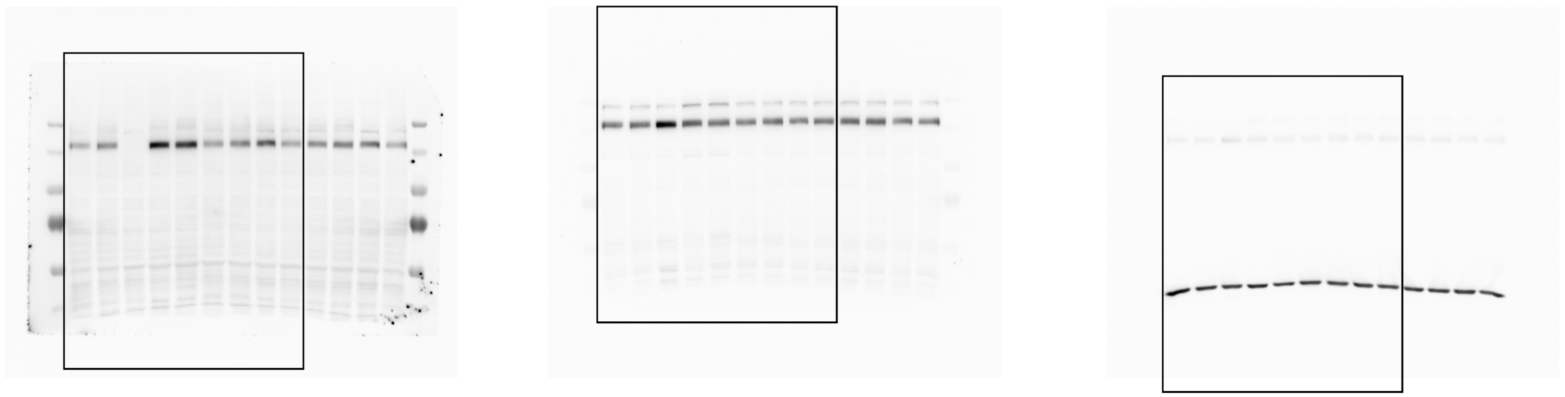

Supplemental Figure 2E

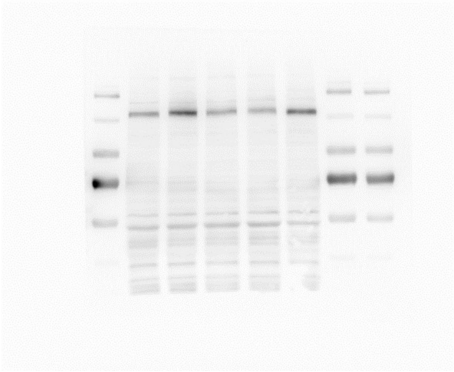

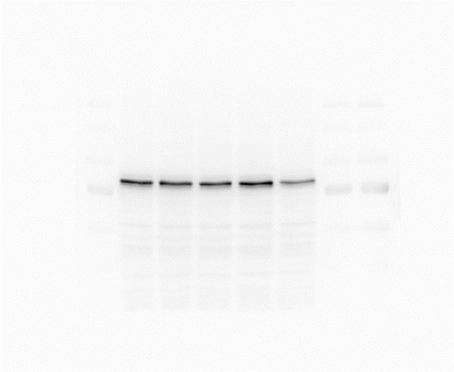

Supplemental Figure 3D

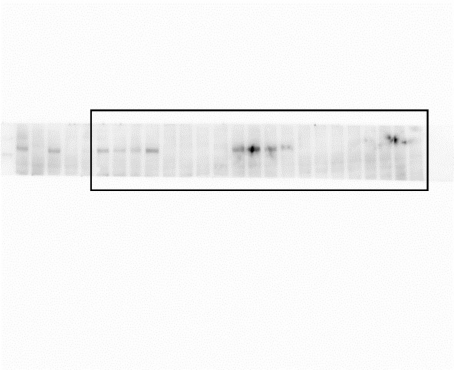
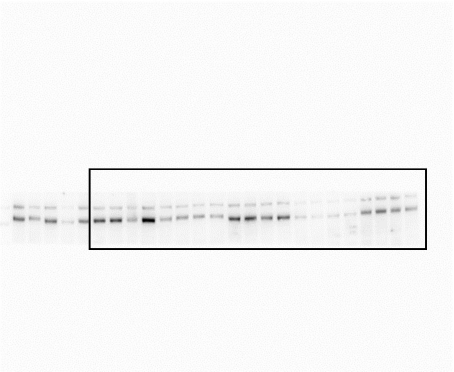
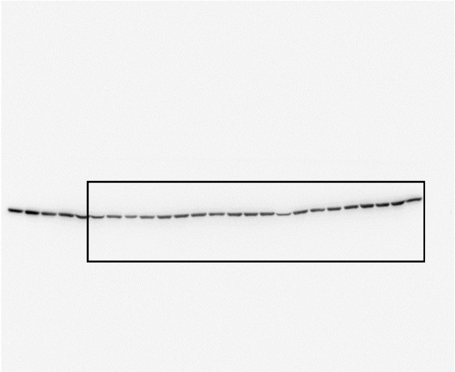

Supplemental Figure 6B

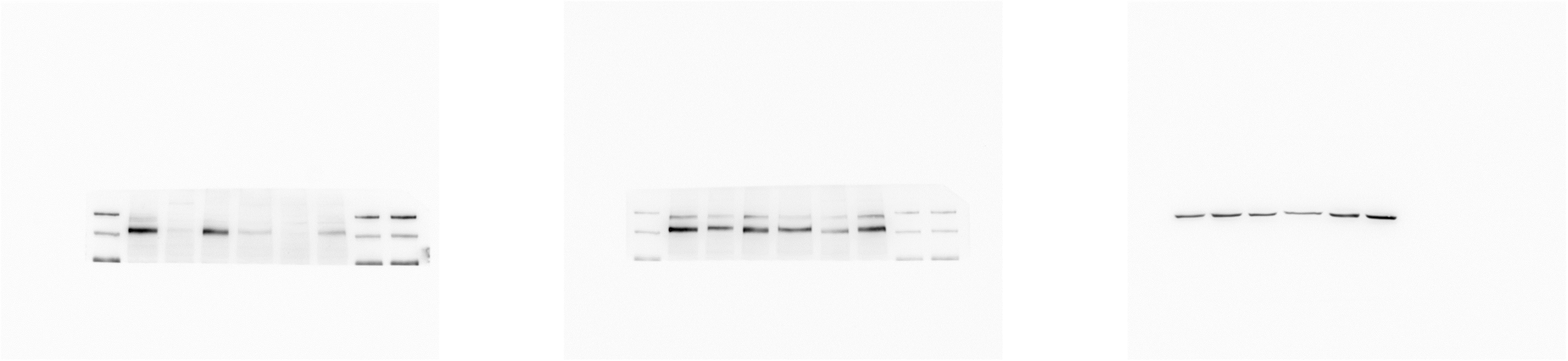

Supplemental Figure 6C
