# Supplementary figures and images for: Targeting the Met-RIPK1 signaling axis to enforce apoptosis and necroptosis in colorectal cancer
Source: Cell Death Dis. 2025 Oct 20;16(1):733. doi: 10.1038/s41419-025-08054-5 (PMC12537949; doi:10.1038/s41419-025-08054-5)

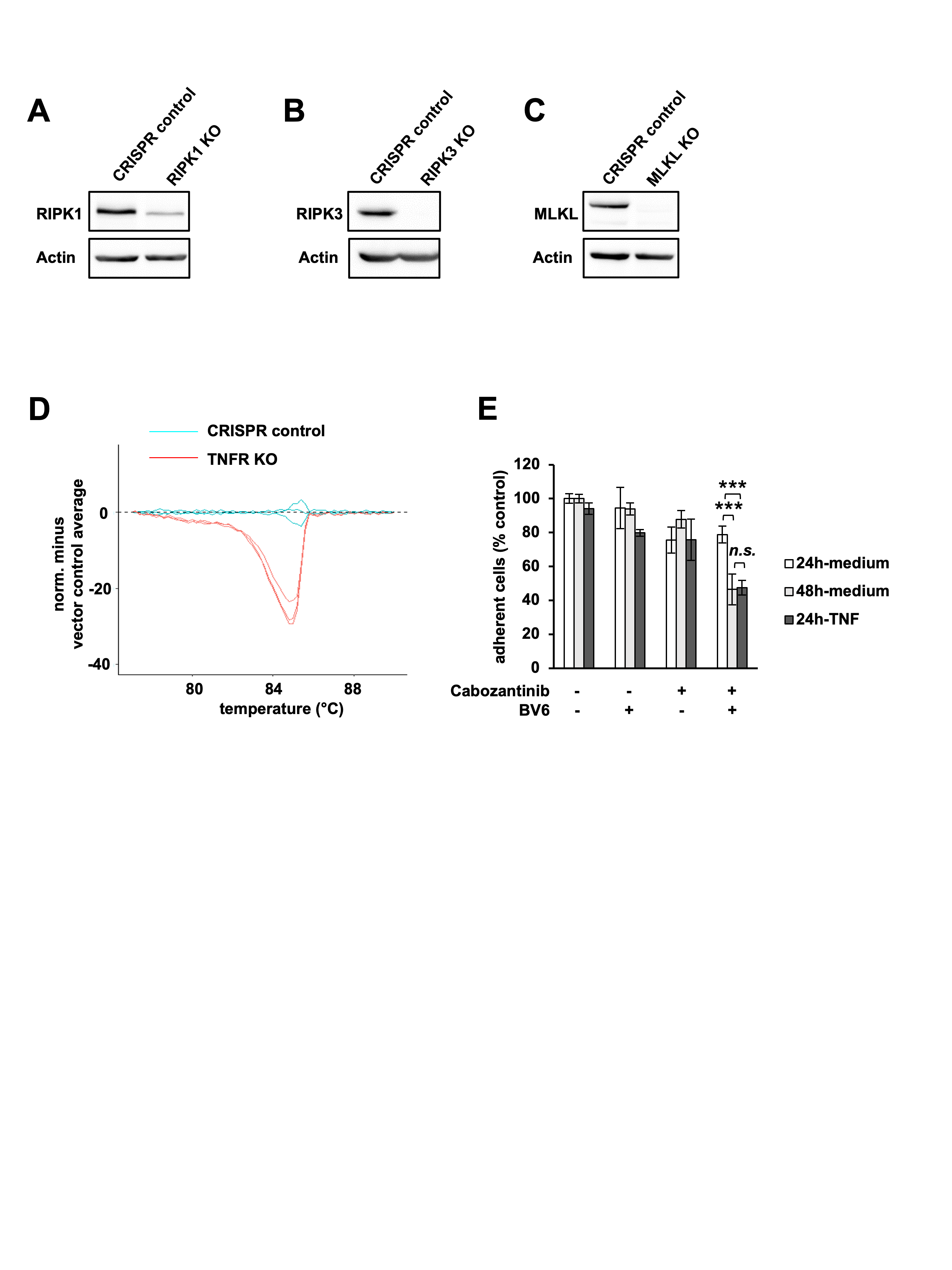

Supplement: Supplementary file 3 — Supplemental Figure 1 [file 41419_2025_8054_MOESM3_ESM.tif]

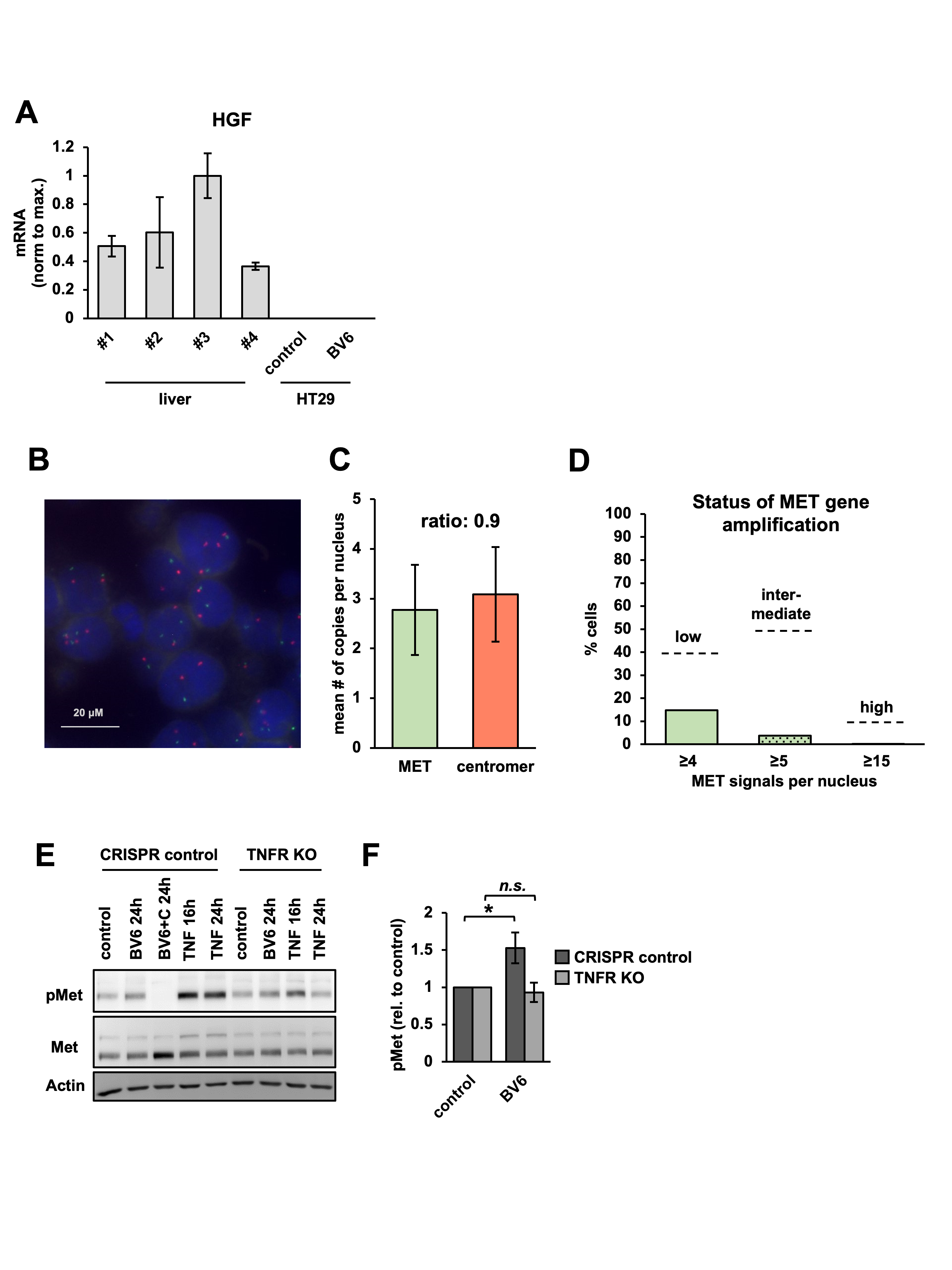

Supplement: Supplementary file 4 — Supplemental Figure 2 [file 41419_2025_8054_MOESM4_ESM.tif]

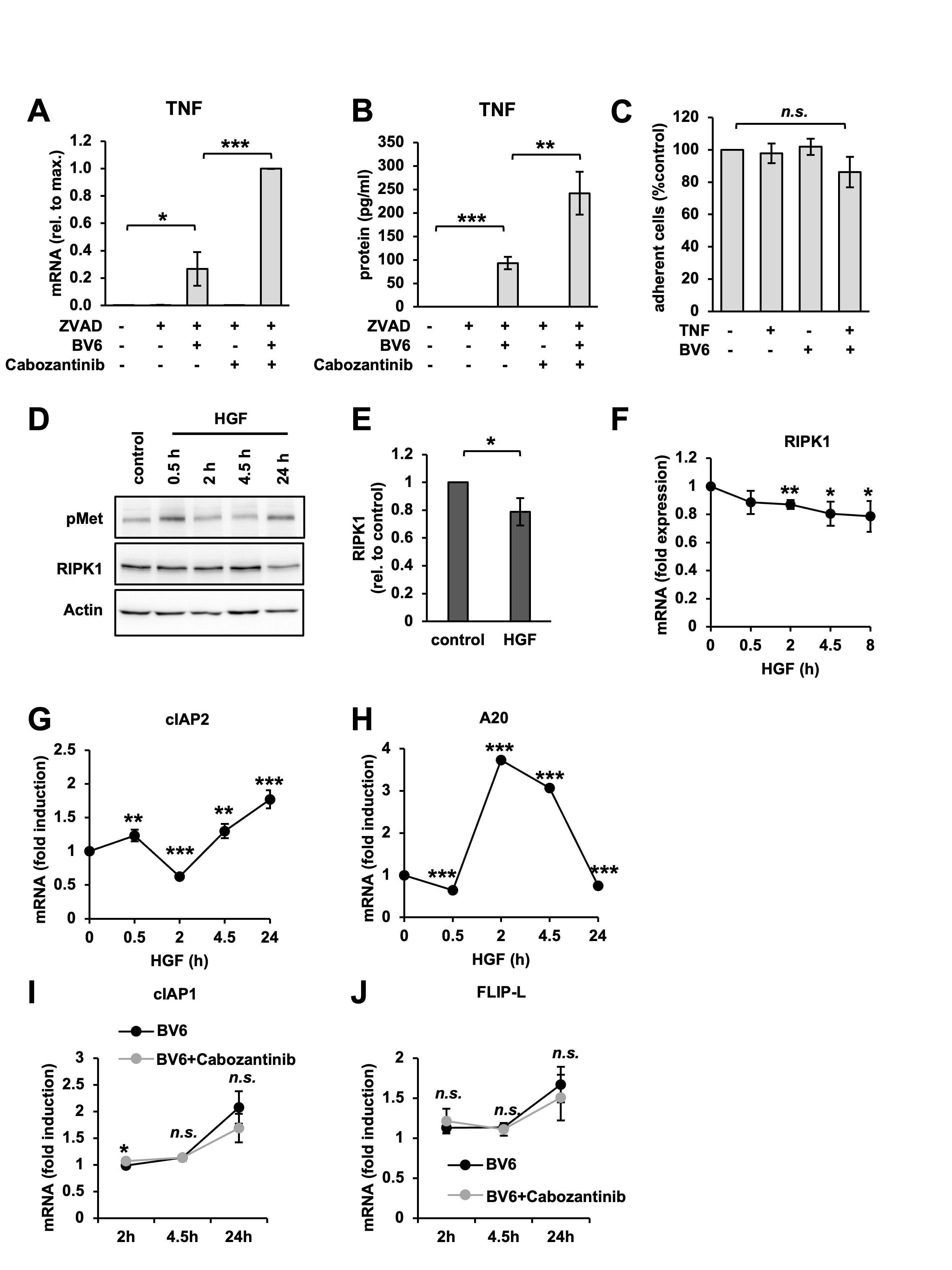

Supplement: Supplementary file 5 — Supplemental Figure 3 [file 41419_2025_8054_MOESM5_ESM.tif]

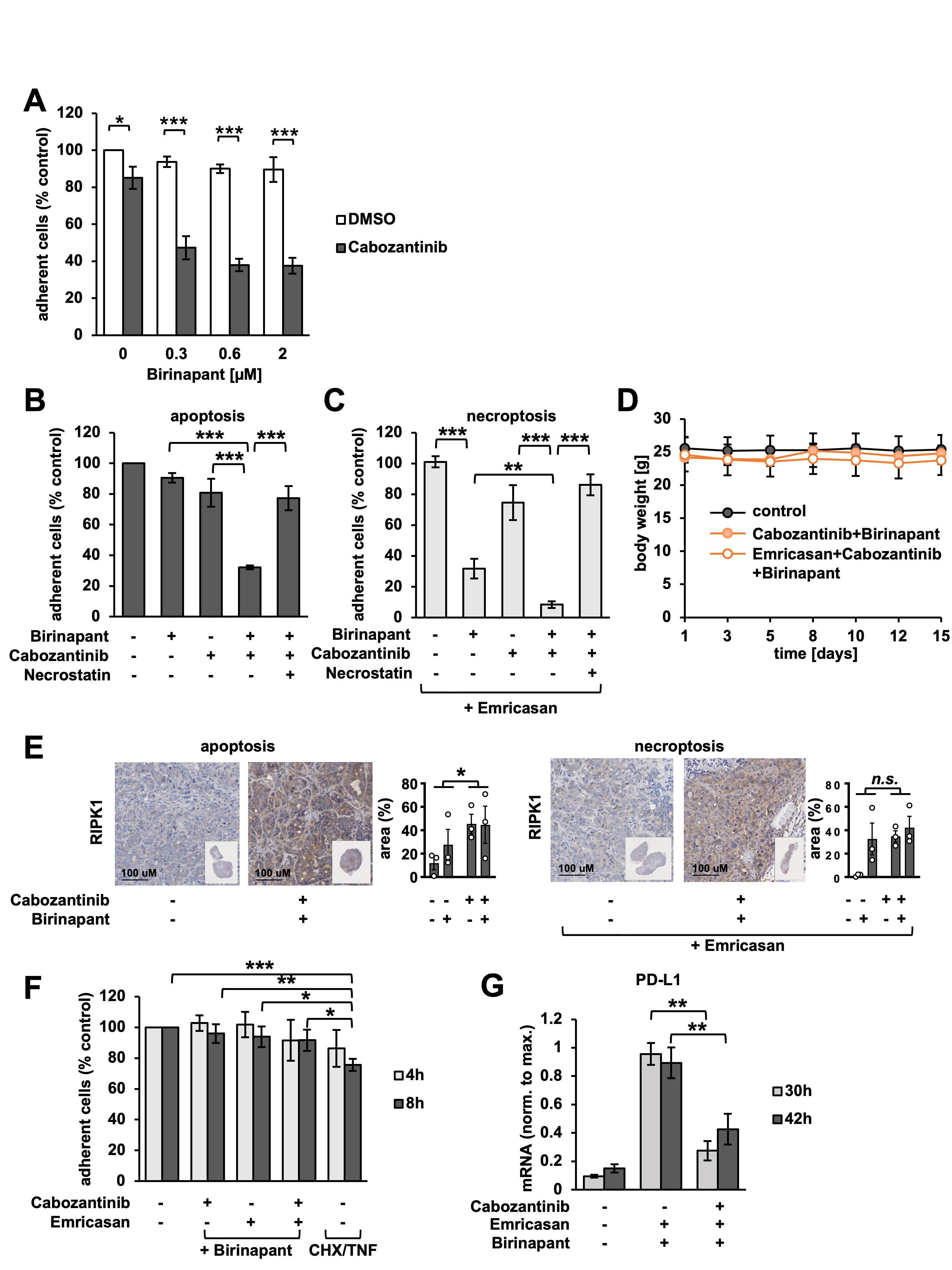

Supplement: Supplementary file 6 — Supplemental Figure 4 [file 41419_2025_8054_MOESM6_ESM.tif]

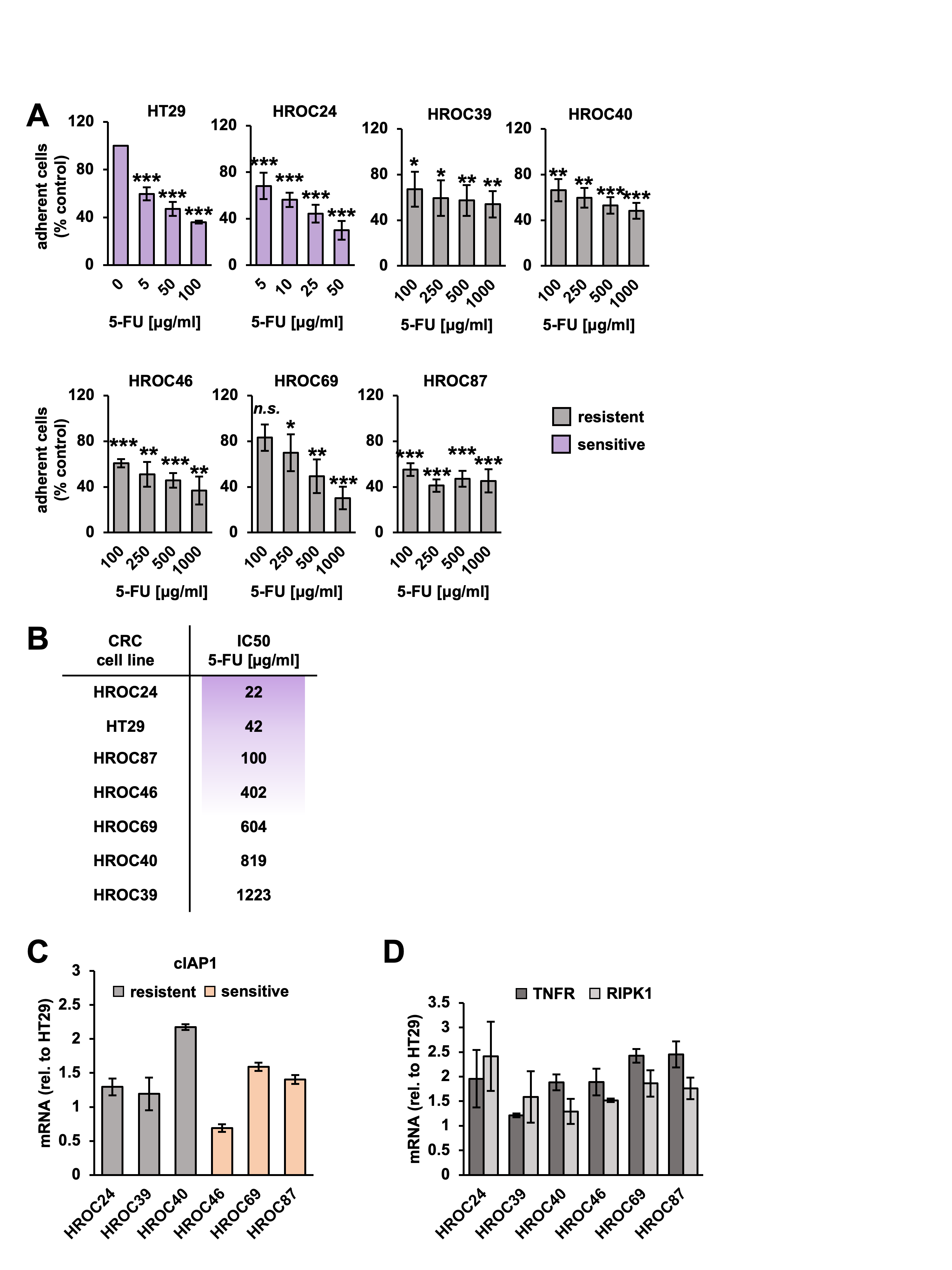

Supplement: Supplementary file 7 — Supplemental Figure 5 [file 41419_2025_8054_MOESM7_ESM.tif]

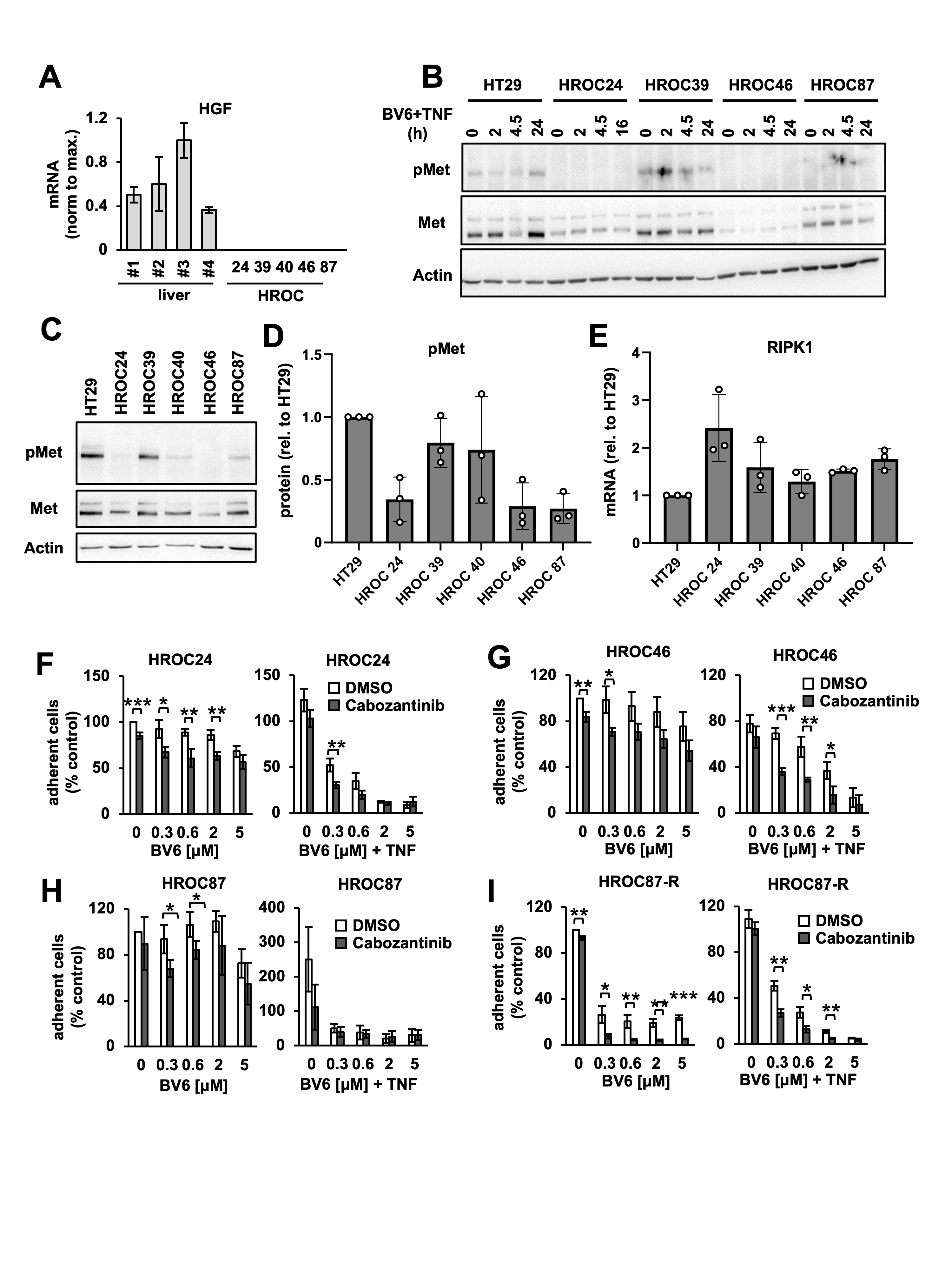

Supplement: Supplementary file 8 — Supplemental Figure 6 [file 41419_2025_8054_MOESM8_ESM.tif]
